# A Computational Study on the Atmospheric Fate of Carbon-Centered Radicals from the 3‑Methyl-2-butene-1-thiol + ^•^OH Reaction: Mechanistic Insights and Atmospheric Implications

**DOI:** 10.1021/acs.jpca.5c00743

**Published:** 2025-07-18

**Authors:** Parandaman Arathala, Avinash Kumar, Rabi A. Musah

**Affiliations:** † Department of Chemistry, 5779 Louisiana State University, Baton Rouge, Louisiana 70803, United States; ‡ Aerosol Physics Laboratory, Physics Unit, Faculty of Engineering and Natural Sciences, 7840 Tampere University, 33720 Tampere, Finland; ⊥ Department of Chemistry, University at Albany-State University of New York, 1400 Washington Avenue, Albany, New York 12222, United States

## Abstract

The reaction of 3-methyl-2-butene-1-thiol (MBT; (CH_3_)_2_C=CHCH_2_SH) with the OH radical is reported to proceed via the addition to either of the sp^2^ hybridized C atoms, forming the two distinct C-centered radicals: (CH_3_)_2_C­(OH)­C^•^HCH_2_SH (R1) and (CH_3_)_2_C^•^CH­(OH)­CH_2_SH (R2). Understanding the fate of these radicals is important for elucidating MBT’s atmospheric transformation mechanisms and the reaction products. Using quantum chemical calculations and kinetic modeling, we show that the unimolecular dissociation as well as isomerization reactions of R1 are kinetically unfavorable due to high energy barriers, and that R1 most likely reacts with atmospheric O_2_ to form R1O_2_ ((CH_3_)_2_C­(OH)­CH­(OO^•^)­CH_2_SH). In contrast, R2 can either undergo isomerization to form the sulfur-centered MBT–OH radical or add O_2_ to form R2O_2_ ((CH_3_)_2_C­(OO^•^)­CH­(OH)­CH_2_SH). These radicals undergo HO_2_ elimination and intramolecular hydrogen atom transfer (HAT) pathways. Specifically, intramolecular HAT from the –SH group to the terminal oxygen atom of R–OO forms S-centered QOOH radicals, with barrier heights of −18.6 and −18.3 kcal mol^–1^ for R1O_2_ and R2O_2_, respectively, calculated relative to those of the R1 + O_2_ and R2 + O_2_ reactants. Rate coefficients for key pathways, including unimolecular dissociation and O_2_ addition followed by subsequent reactions, were calculated and analyzed. The kinetics results suggest that the intramolecular H atom transfer paths of R1O_2_ and R2O_2_ are significantly faster by ∼3 orders of magnitude compared to their bimolecular reactions with NO/HO_2_, respectively. The findings suggest that under low NO concentrations R1O_2_ and R2O_2_ are capable of undergoing H-shift-driven autoxidation mechanisms. The atmospheric implications are discussed. Results indicate that MBT-derived peroxy radicals contribute to tropospheric chemistry by generating reactive species such as highly oxygenated peroxy radicals, HC­(O)­CH_2_SH, (CH_3_)_2_C­(OH)­C­(=O)­H, CH_3_C­(O)­CH_3_, and various S- and C-centered alkyl radicals in the atmosphere.

## Graphical abstract

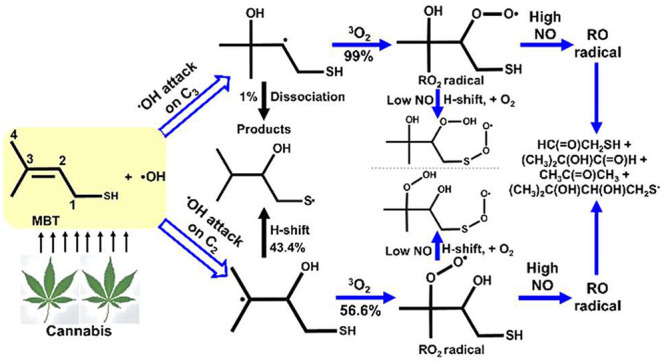

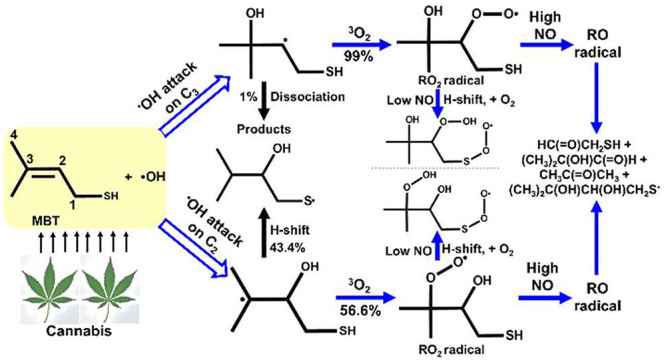

## 1 Introduction

The chemistry of volatile organosulfur compounds (VOSCs) is important because their transformation in the atmosphere can affect air quality and climate change.^1,2^ In addition, the global sulfur budget is of major interest due to the need to assess the contribution of biogenic sulfur emissions that is required to balance the global sulfur cycle.^3^ In this regard, the origins and removal processes of sulfur compounds produced by terrestrial organisms remain the most significant uncertainty in our understanding of the global sulfur cycle. More precisely, emission of sulfur compounds from live vascular plants and their transformation mechanisms in the atmosphere have not been fully characterized.^4^

Among the catalog of terrestrial plant-derived organosulfur compounds is 3-methyl-2-butene-1-thiol (MBT; (CH_3_)_2_C=CHCH_2_SH), a molecule emitted by both hemp and marijuana varieties of .^5,6^ It is believed to contribute to the plant’s skunky odor. While there are no reports on the ambient concentration of MBT in the atmosphere, occupies an increasing amount of farmland. For example, the United States was recognized as the world’s largest producer of industrial hemp with a licensed cultivation area covering 465,787 acres in 2020.^7^ Alongside the U.S., China and Canada were leading countries in global industrial hemp cultivation in 2019.^8^ This indicates significant acreage dedicated to *Cannabis* cultivation both in the U.S. and globally. Given the possible large-scale implementation of *Cannabis* cultivation, it is likely that there will be relatively significant discharges of MBT into the atmosphere.

Currently, a complete understanding of the atmospheric chemistry and environmental risk of MBT is unclear. Recently, we reported the primary oxidation of MBT by hydroxyl radicals (^•^OH) using high-level computational calculations and chemical kinetic analysis.^9^ The rate coefficients were calculated for all possible addition and abstraction pathways. The results suggested that the primary transformation of MBT occurs through OH radical addition rather than H-abstraction pathways. The branching ratios indicated that ∼85–92% of the overall reaction at temperatures between 200 and 298 K proceeds via an addition mechanism. Figure 1 presents two addition pathways for the reaction of the MBT + OH radical.^9^ The reaction shown in eq 1 suggests OH addition to the C_3_ carbon, which results in the formation of the (CH_3_)_2_C­(OH)­C^•^HCH_2_SH (R1) product, with a branching ratio of ∼72–87%. In contrast, the reaction shown in eq 2 indicates OH addition at the C_2_ carbon atom, leading to the formation of the (CH_3_)_2_C^•^CH­(OH)­CH_2_SH (R2) radical product, which contributes ∼5–11% to the overall reaction. These reported branching ratios highlight the significance that the addition paths play, in contrast to the abstraction paths, in the MBT + OH radical reaction. The lifetimes (τ) of MBT with respect to its reaction with the OH radical in the Earth’s tropospheric temperatures between 200–298 K were reported to be only 2.0–5.0 h. We believe that the significant emissions and short lifespan of MBT may hint at a more pronounced effect on the local environment where it appears. Therefore, to further explore the atmospheric transformation of MBT, it is crucial to investigate the fate of the major products formed through the addition paths associated with the MBT + OH radical reaction, which is essential for assessing the global chemical impact of the radical products that are first generated when MBT is intercepted by the hydroxyl radical.

**Figure 1:**
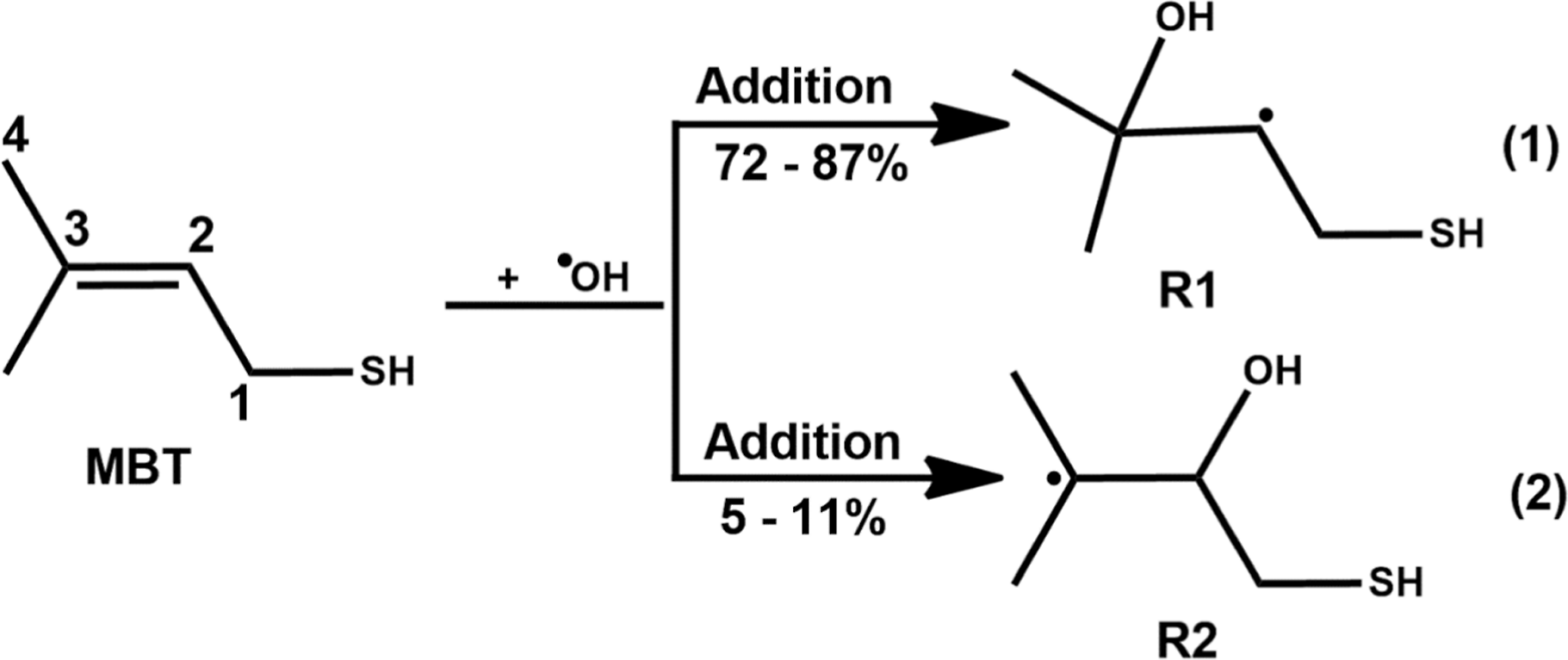
OH radical attack at the C_3_ and C_2_ carbon atoms of MBT, leading to the formation of carbon-centered radicals R1 and R2, respectively. The symbols R1 and R2 represent (CH_3_)_2_C­(OH)­C^•^HCH_2_SH and (CH_3_)_2_C^•^CH­(OH)­CH_2_SH, respectively.

Once formed, the carbon-centered radicals R1 and R2 can undergo either self-dissociation or react further with molecular oxygen (O_2_) to form MBT–OH-derived peroxy radicals (RO_2_) in the atmosphere. Such RO_2_ radicals play a crucial role in atmospheric chemistry, particularly influencing air quality and the formation of secondary pollutants.^10^ Previous studies indicate that the fate of RO_2_ radicals depends on two competing reaction pathways: unimolecular rearrangements and bimolecular reactions with NO and HO_2_ radicals.^10–13^ Unimolecular rearrangements, specifically rapid intramolecular hydrogen atom transfers (HATs), have been observed in various RO_2_ radicals. For instance, HATs have been identified in RO_2_ radicals generated during the oxidation of isoprene and other compounds.^14–18^ These rapid HAT processes in RO_2_ radicals facilitate the autoxidation of VOCs under low-NOx conditions, leading to the production of highly oxygenated molecules (HOMs).^19–23^ HOMs, in turn, play a crucial role in the formation of secondary organic aerosols and contribute to new particle formation.^24,25^

In this study, we sought to better understand the atmospheric behavior and environmental implications of MBT emitted into the environment by investigating the fate of the carbon-centered radicals R1 and R2 (see Figure 1) that are formed from its initial reaction with a hydroxyl radical. Investigated reactions, performed by high-level computational methods combined with kinetic rate calculations, included their self-dissociation, subsequent reactions with O_2_, and further reactions with NO and HO_2_ radicals. The mechanisms of isomerization and dissociation of carbon-centered radicals R1 and R2, as well as their reactions with O_2_, are crucial for understanding both their atmospheric chemistry and associated environmental risks. For their self-isomerization and dissociation reactions, we characterized not only all the minima and transition states on the PES profiles but also the minima and transition states for subsequent reactions with O_2_ using high-level computational calculations. We also determined the kinetic parameters for various possible reaction paths under atmospheric conditions. The results enhance understanding of MBT-related RO_2_ chemistry and offer valuable information for air quality modeling and policy development to mitigate air pollution levels that pose risks to human health.

## 2 Computational and Kinetics Methodology

The geometries of all of the reactants, transition states, and intermediates identified on the PES profiles for self-dissociation of carbon-centered radicals R1 and R2, followed by subsequent reactions of these radicals initiated by O_2_ were optimized, and harmonic frequency calculations were performed using density functional theory (M06-2X)^26^ and the aug-cc-pV­(T+d)­Z basis set. The M06-2X functional is suggested for use in calculating thermochemical, kinetics, and noncovalent interactions due to its strong performance across a wide range of reactions relevant in the atmosphere.^27–29^ Using the aug-cc-pV­(T+d)­Z basis set offers the benefit of incorporating additional tight d-functions, which provide a more accurate representation of bonding in compounds containing sulfur atoms.^30,31^ All of the optimized intermediates and transition states were validated by finding only the real harmonic vibrational frequency and one imaginary vibrational frequency, respectively. The intrinsic reaction coordinate (IRC) computations were performed at the same M06-2X/aug-cc-pV­(T+d)­Z level to verify that all the obtained transition states connected to their respective pre- and postreactive complexes on the reaction coordinate.^32^ The M06-2X calculations were performed using the Gaussian 16 software suite, following the standard convergence criteria.^33^ The energies of the reactants, intermediates, transition states, and products were further refined by recalculating their single-point electronic energies at the RHF-RCCSD­(T)-F12a/VDZ-F12 level using the Molpro 2022.2.2 program.^34^ The RHF-RCCSD­(T)-F12a/VDZ-F12 method offers high accuracy and has shown very good agreement with higher-level results, along with much faster basis set convergence through the RHF-RCCSD­(T)-F12a/VDZ-F12 approach.^35^ Additionally, the combination of RHF-RCCSD­(T)-F12a/VDZ-F12 and M06-2X/aug-cc-pV­(T+d)­Z levels has been employed by other research groups to investigate the energetics and kinetics of reactions between O_2_ and atmospherically relevant radicals.^13,19^ Accordingly, we adopted a similar methodology in our study. We obtained the final energies for each stationary point with the RHF-RCCSD­(T)-F12a/VDZ-F12 energies and the zero-point correction to the energy from the M06-2X/aug-cc-pV­(T+d)­Z level calculations. In all RHF-RCCSD­(T)-F12a/VDZ-F12 calculations, the T1 diagnostic was below 0.03, indicating that the influence of multireference character was minimal.^36^

The rate coefficients for unimolecular dissociation of C-centered radicals R1 and R2, and their subsequent reactions with atmospheric O_2_, were determined using the Master equation solver for multi-energy well reactions (MESMER) kinetic code,^37^ a program widely employed to investigate the kinetics of reactions involving atmospheric compounds.^27,38–41^ The Mesmer ILT method was used for the formation of barrierless peroxy radical adducts (RO_2_) on the PES. The Rice–Ramsperger–Kassel–Marcus (RRKM) method was applied to calculate the rate coefficients for RO_2_ unimolecular reactions with a well-characterized transition state. In the Mesmer ILT approach, a temperature-independent capture rate coefficient of 6 × 10^–12^ cm^3^ molecule^–1^ s^–1^ was used.^39,42,43^ This value is comparable to experimental rates of typical alkyl + O_2_ reactions at 298 K.^44^ The modified Arrhenius parameter of −0.5 and an activation energy of 0 kcal mol^–1^ were assumed. MESMER input parameters, such as rotational constants, vibrational frequencies, and energies for all stationary points on the PESs, are required for kinetic modeling. These were derived from the present M06-2X/aug-cc-pV­(T+d)­Z level calculations and ZPE corrected energies at the RHF-RCCSD­(T)-F12a/VDZ-F12//M06-2X/aug-cc-pV­(T+d)­Z level. Nitrogen (N_2_) was utilized as the buffer gas in these simulations. The average downward collision energy transfer (i.e., *E*_down_) was considered in the MESMER calculations. Using the exponential-down model, a value of 200 cm^–1^ was employed for collision between reactive intermediates and the bath gas N_2_. The Lennard-Jones parameters for all the intermediates were approximated using values for an alkane of a size similar to the intermediates involved in the various elimination and HAT paths in this study (i.e., *n*-hexane: ε = 201 K, σ = 4.4 Å).^45^ The potential impact of tunneling effects on the rate coefficient calculations, particularly for processes like intramolecular hydrogen atom transfer and HO_2_ elimination, was modeled using a one-dimensional Eckart barrier approach.^46^ The MESMER input files for all of the rate coefficients calculated for the reactions studied in the present work are provided in the Supporting Information. The RRKM-ME calculations using MESMER utilize the lowest-lying energy conformers for the R1O_2_ and R2O_2_ radicals and all other possible transition states for all of the studied reactions. Because MESMER code does not support multiconformer analysis, we employed the multiconformer transition state theory (MC-TST) approach^47,48^ to calculate the rate coefficients for the major H atom transfer reactions via TS25 and TS35, leading to the formation of the corresponding S-centered QOOH radicals in both the R1O_2_ and R2O_2_ radical systems.

## 3 Results and Discussion

### 3.1 Self-Dissociation and Isomerization of R1 and R2 Radicals

Investigation of the fate of the major carbon-centered MBT–OH radicals (R1 and R2) formed through the addition paths in the reaction of the MBT + OH radical (see reactions shown in eqs 1 and 2 in Figure 1) was conducted to understand the transformation mechanism of MBT under atmospheric conditions. Based on previously reported energy and kinetic data, the R1 and R2 radicals are the dominant products of the MBT + OH radical reaction, releasing 30.5 and 28.4 kcal mol^–1^ of energy, respectively.^9^ In principle, the chemically activated R1 and R2 radicals can undergo self-isomerization and dissociation under atmospheric conditions. Possible self-isomerization and dissociation reactions of R1 and R2 leading to various products are shown in Figures 2 and 3, and their corresponding PES profiles are displayed in Figures 4 and 5, respectively. According to Figure 2, R1 can undergo several dissociation reactions via eqs 3–7 and several self-isomerizations via eqs 8–11, to form the respective dissociation and isomerization products. Similarly, Figure 3 indicates R2 can undergo dissociation via eqs 12–16 to form their corresponding dissociation products and self-isomerizations via eqs 17–21 to form isomerization products. The energies of all the stationary points on the potentials were calculated at the RHF-RCCSD­(T)-F12a/VDZ-F12//M06-2X/aug-cc-pV­(T+d)­Z level and are presented in Figures 4 and 5. The optimized structures of the transition states for the various dissociation and isomerization reactions for R1 and R2 are shown in Figure S1. The results in Figure 4 indicate that the barrier heights for the dissociation (see eqs 3–6 in Figure 2) and isomerization reactions (eqs 8–11 in Figure 2) of R1 are greater than 18.7 kcal mol^–1^, which suggests that the self-dissociation and isomerization reactions have significantly larger barriers and proceed slowly in the atmosphere. Interestingly, we found that the barrier height for the dissociation reaction shown in eq 7, involving C–S single bond scission concomitant with the formation of a double bond between carbon atoms (through TS5), leading to the formation of ^•^SH and P_5_ ((CH_3_)_2_C­(OH)­CH=CH_2_), was 9.3 kcal mol^–1^ (see Figures 4, 2, and S1). This value is ∼9.4–40.3 kcal mol^–1^ below the barrier heights of other possible channels. Therefore, dissociation of R1 to form ^•^SH + P_5_ through eq 7 was found to be dominant compared to other possible channels.

**Figure 2:**
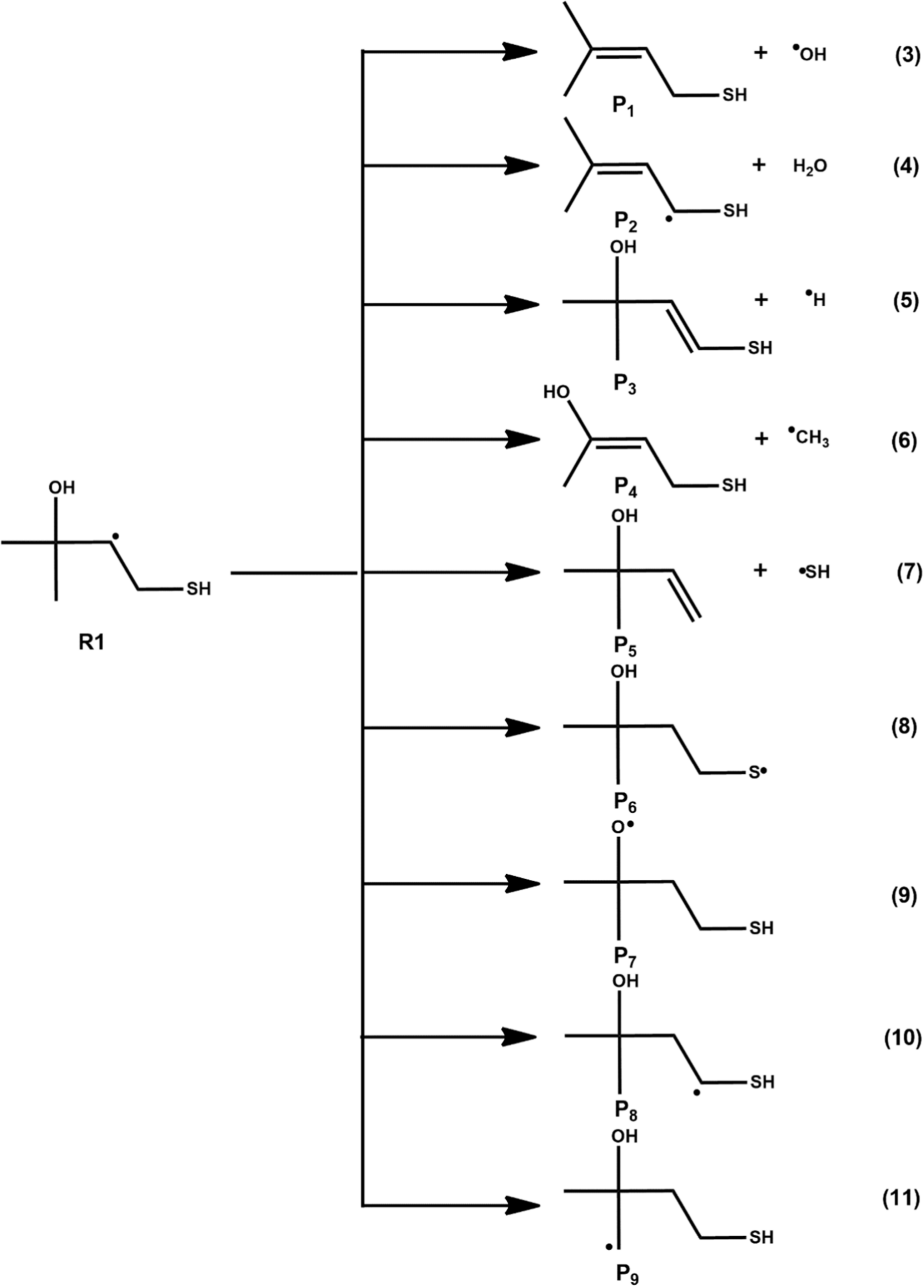
Various possible self-dissociation (eqs 3–7) and isomerization (eqs 8–11) reactions of carbon-centered radical R1. The symbols P_*n*_ (*n* = 1–9) represent products.

**Figure 3:**
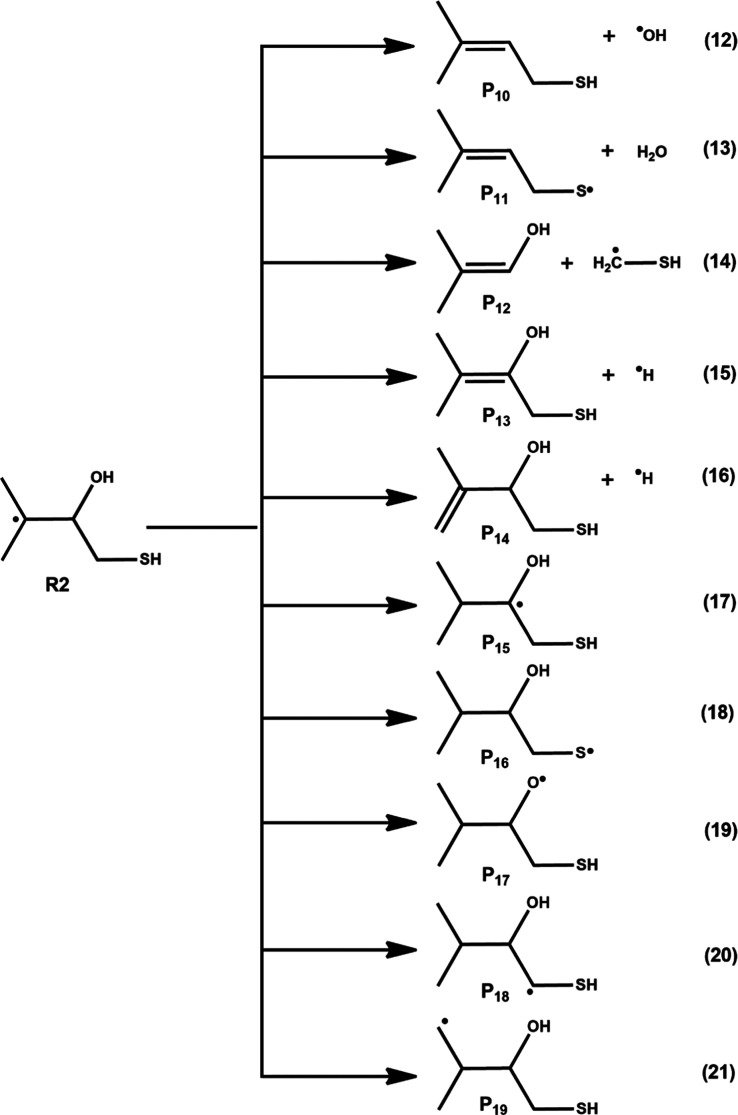
Various possible self-dissociation (eqs 12–16) and isomerization (eqs 17–21) reactions of carbon-centered radical R2. The symbols P_*n*_ (*n* = 10–19) represent products.

**Figure 4:**
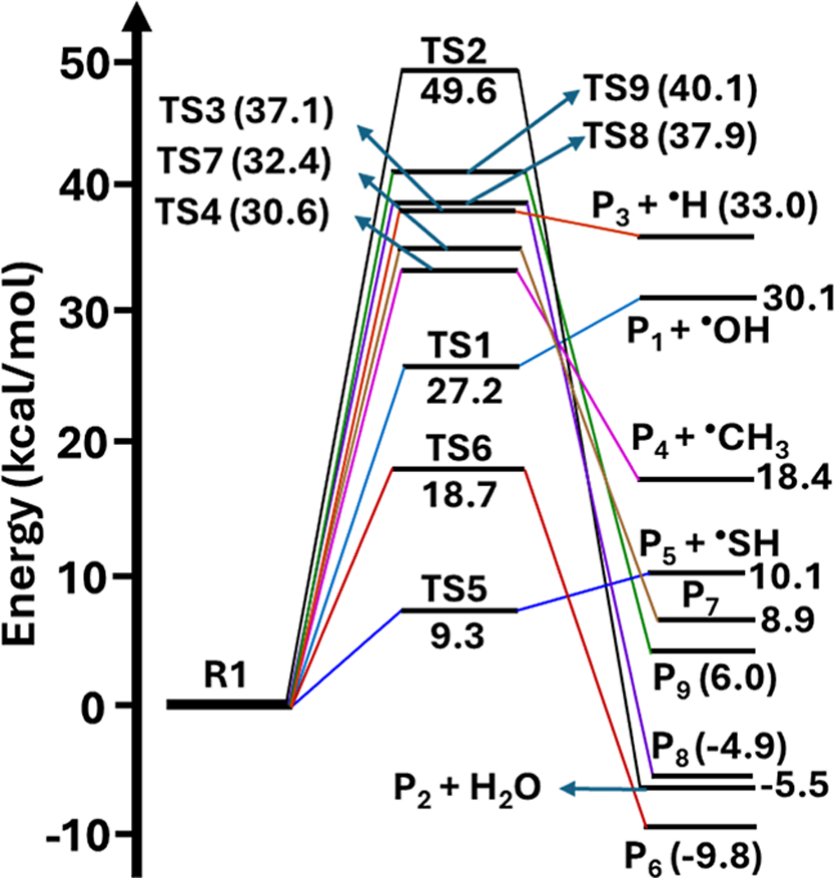
The RHF-RCCSD­(T)-F12a/VDZ-F12//M06-2X/aug-cc-pV­(T+d)­Z level calculated potential energy surface profiles for self-isomerization and dissociation paths of carbon-centered radical R1. The symbols R1, TS1-TS9, and P_1_–P_9_ represent (CH_3_)_2_C­(OH)–C^•^HCH_2_SH, transition states, and products, respectively.

**Figure 5:**
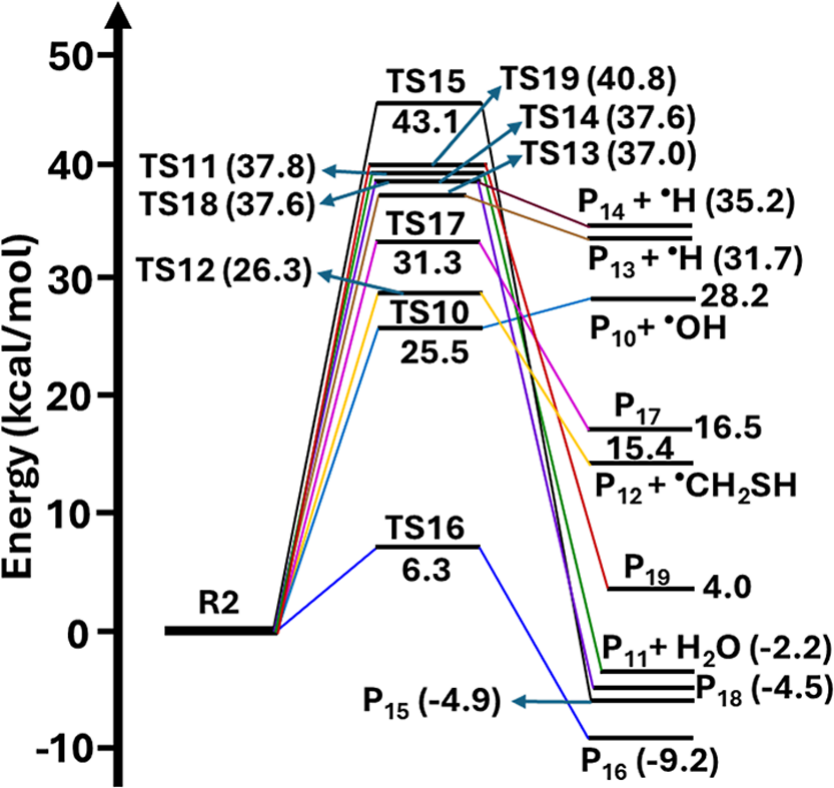
The RHF-RCCSD­(T)-F12a/VDZ-F12//M06-2X/aug-cc-pV­(T+d)­Z level calculated potential energy profiles for self-isomerization and dissociation paths of carbon-centered radical R2. The symbols R2, TS10-TS19, and P_10_–P_19_ represent (CH_3_)_2_C^•^–CH­(OH)­CH_2_SH, transition states, and products, respectively.

Similarly, the PES profiles in Figure 5 show that the barrier heights for all the self-dissociation (eqs 12–16) and isomerization (eq 17, eqs 19–21) channels (see Figure 3) are greater than 25.5 kcal mol^–1^. This indicates that these reactions have significantly high barriers and are unlikely to occur under atmospheric conditions. However, the barrier height for the self-isomerization of C-centered radical R2 via TS16 by 1,4-H atom transfer from the –SH group to the radical center on the carbon atom (see Figure S1) was found to be 6.3 kcal mol^–1^. This results in formation of a S-centered MBT–OH radical product (P_16_; (CH_3_)_2_CHCH­(OH)­CH_2_S^•^) (see Figure 5 and eq 18 in Figure 3). This indicates that the barrier height for this reaction is ∼19.2–36.8 kcal mol^–1^ lower compared to the barrier height values of all other channels, making it predominant.

The present results suggest that the self-isomerization and dissociation of R1 and R2 generally involve high energy barriers and proceed slowly under tropospheric conditions with the exception of the isomerization of carbon-centered radical R2 to form P_16_ (refer to the rate coefficient data for the most dominant reactions of R1 and R2 in the kinetics section). Rather, due to the high concentration of triplet oxygen molecules in the atmosphere, the two carbon-centered radicals R1 and R2 rapidly react with atmospheric O_2_, forming the respective alkyl peroxyl radicals (RO_2_). Subsequent intramolecular hydrogen atom transfer (HAT) reactions of the RO_2_ radicals are key for rapid autoxidation and the formation of highly functionalized products. For example, the reaction of R1 + O_2_ and R2 + O_2_ lead to formation of R1O_2_ ((CH_3_)_2_C­(OH)­CH­(OO^•^)­CH_2_SH) and R2O_2_ ((CH_3_)_2_C­(OO^•^)­CH­(OH)­CH_2_SH), respectively. The R1O_2_ and R2O_2_ radical structures suggest that the addition of atmospheric O_2_ occurs at the C-site of the R1 and R2 radicals. We conducted a conformational analysis of each RO_2_ radical and the transition states of major reactions to identify the most stable structures by sampling their conformers using the Spartan’20 (Wave function, Inc.) program.^49^ The conformer sampling was performed using the Merck Molecular Force Field (MMFF) method.^50^ Initially, all conformers were optimized at the B3LYP/6-31+G­(d) level of theory. Conformers with relative electronic energies within 2 kcal mol^–1^ of the lowest-energy structure were then selected for further optimization at the M06-2X/aug-cc-pV­(T+d)­Z level of theory. The fully optimized most stable conformers of the R1O_2_ and R2O_2_ radical adducts are shown in Figure 6. The binding energies of R1 and R2 with O_2_ were found to be ∼−32.5 and −36.7 kcal mol^–1^, respectively, calculated with respect to their corresponding starting reactants at the RHF-RCCSD­(T)-F12a/VDZ-F12//M06-2X/aug-cc-pV­(T+d)­Z level of theory. We concluded that the presence of several hydrogen bonds (see Figure 6) in the RO_2_ radical structures is the main reason for its high stability, although the experimentally reported energies for the formation of these RO_2_ radical adducts are not available. But the present calculated values are 0.5 and 3.7 kcal mol^–1^ higher and lower for R1O_2_ and R2O_2_ radical adducts respectively, than the binding energy of −33.0 kcal mol^–1^ reported at the RCCSD­(T)/6-311+G­(3df,2p) level of theory for the formation of HOCH_2_CH_2_OO^•^ from the reaction ^•^CH_2_CH_2_OH + O_2_.^51^

**Figure 6:**
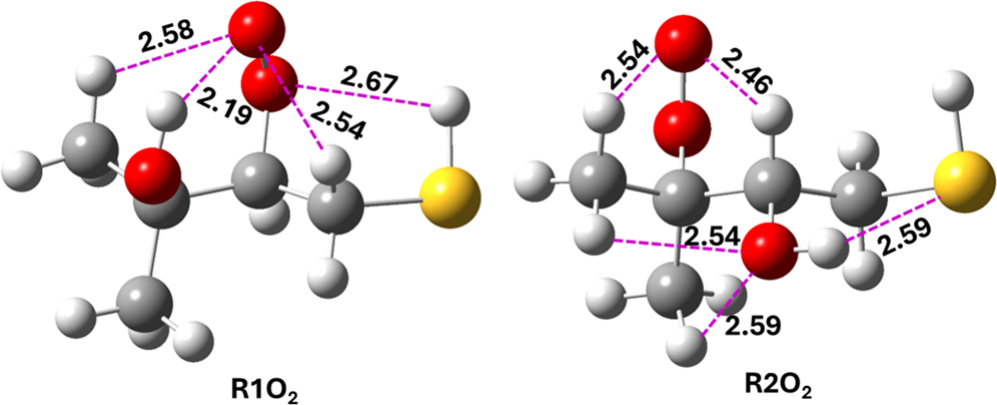
The most stable conformers of RO_2_ radical adducts (R1O_2_ and R2O_2_) formed from the reaction of R1 + O_2_ and R2 + O_2_, optimized at the M06-2X/aug-cc-pV­(T+d)­Z level. The hydrogen bonds are shown with dashed lines and bond lengths are given in Å.

### 3.2 Unimolecular Reactions of the R1O_2_ Radical

A schematic representation of PES profiles for the reaction of R1 + O_2_ leading to the formation of the R1O_2_ radical followed by various elimination paths to form several products is shown in Figure 7. The geometries of the possible transition states and product complexes optimized at the M06-2X/aug-cc-pV­(T+d)­Z level are shown in Figures 7 and S2. The energies of the stationary points on the PES’s were calculated at the RHF-RCCSD­(T)-F12a/VDZ-F12//M06-2X/aug-cc-pV­(T+d)­Z level. Results in Figure 7 indicate that the reaction of R1 + O_2_ commences with the formation of a barrierless R1O_2_ radical with an energy of ∼32.5 kcal mol^–1^ below that of the R1 + O_2_ starting reactants. We identified various transformation pathways for the R1O_2_ radical adduct which are illustrated in Figure 8. It shows that the R1O_2_ radical adduct can undergo two major transformation pathways: (1) elimination and (2) intramolecular HATs. The elimination of HO_2_, ^•^OH, H_2_O, and H_2_S results in the formation of P_20_, P_21_, P_22_, P_23_, and P_24_ products through eqs 22–26. On the other hand, intramolecular HATs can occur from the –SH, –CH_2_, –CH_3_, and –OH moieties of R1O_2_ to the terminal O atom of the R–OO group, forming the corresponding sulfur-, carbon-, and oxygen-centered hydroperoxyalkyl radicals (^•^QOOH). These QOOH radicals are composed of a hydroperoxide (−OOH) group and new S, C, and O radical centers (^•^Q) (see reactions in eqs 27–30 in Figure 8). In principle, another C-centered QOOH radical could form via an intramolecular HAT from the –CH group of R1O_2_ to the terminal O atom of the R–OO group. However, the transition state for this reaction pathway was not observed in our current M06-2X/aug-cc-pV­(T+d)­Z calculations. Instead, our calculations consistently lead to the formation of a transition state involving H atom transfer from the –CH group of R1O_2_ to the terminal oxygen, followed by O–O bond scission, due to the weak O–O bond in the R1O_2_ radical. This process results in the formation of (CH_3_)_2_C­(OH)­C­(=O)­CH_2_SH and an OH radical as the final products. This pathway was considered in the elimination paths of the R1O_2_ radical (see Figure 8).

**Figure 7:**
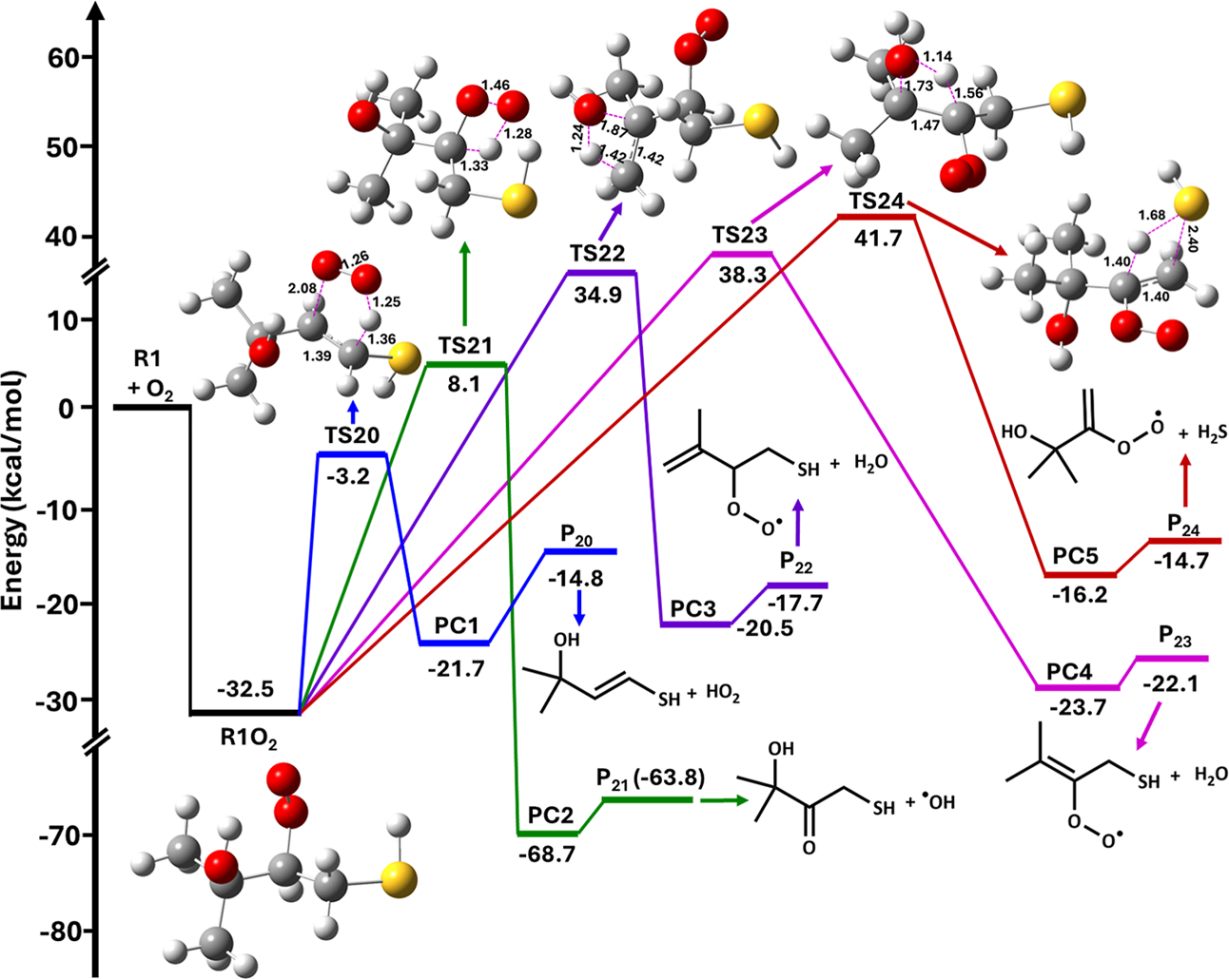
Potential energy profiles for the reaction of R1 + O_2_ leading to the R1O_2_ radical, followed by various possible unimolecular elimination channels to form their respective products, calculated at the RHF-RCCSD­(T)-F12a/VDZ-F12//M06-2X/aug-cc-pV­(T+d)­Z level. R1O_2_ = (CH_3_)_2_C­(OH)­CH­(OO^•^)­CH_2_SH; TS20–TS24 = transition states; PC1-PC5 = postreactive complexes; and P_20_–P_24_ = products.

**Figure 8:**
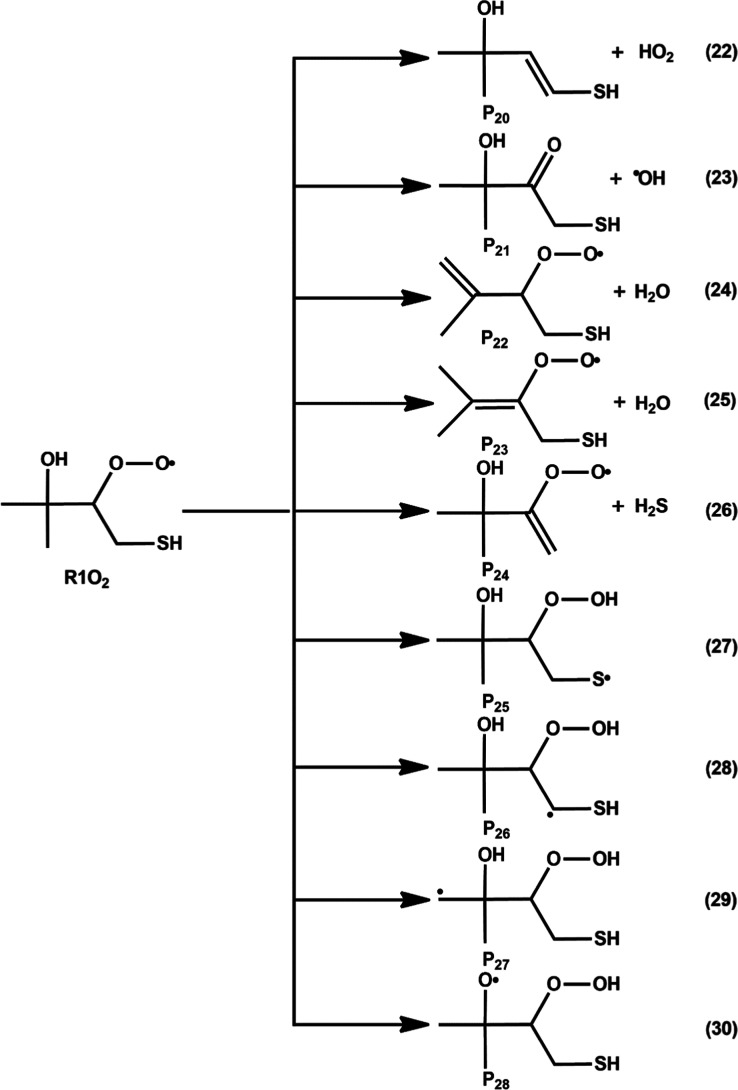
Various possible elimination and HAT pathways for R1O_2_ from the R1 + O_2_ reaction. Equations 22–26 represent elimination paths, and eqs 27–30 show HAT reactions. P_20_–P_28_ represent products.

The PES profiles for the multiple elimination paths for the R1O_2_ reaction obtained at the RHF-RCCSD­(T)-F12a/VDZ-F12//M06-2X/aug-cc-pV­(T+d)­Z level are shown in Figure 7. The barrier height for the elimination of the HO_2_ radical via TS20 was found to be −3.2 kcal mol^–1^ below the R1 + O_2_ starting reactants. This reaction path further proceeds to the stable intermediate PC1 and then to the formation of P_20_ ((CH_3_)_2_C­(OH)­CH=CHSH + HO_2_) on the PES at −14.8 kcal mol^–1^ below that of the starting R1 + O_2_ reactants. Similarly, elimination of the OH radical, H_2_O, and H_2_S occurs from R1O_2_ through TS21, TS22, TS23, and TS24 with their respective barrier heights of 8.1, 34.9, 38.3, and 41.7 kcal mol^–1^ above that of the R1 + O_2_ reactants. These reactions further continue to the formation of their corresponding intermediates (PC2–PC5) and finally form products P_21_, P_22_, P_23_, and P_24_ (see Figure 7). Based on the barrier height results, HO_2_ elimination via TS20 was found to be dominant by virtue of its lower barrier when compared to ^•^OH, H_2_O, and H_2_S elimination via TS21, TS22, TS23, and TS24, all of which have larger barriers and are energetically less feasible.

The PES profiles for intramolecular HAT reactions involving transfer of an H atom from the –SH, –CH_2_, –CH_3_, and –OH groups to the terminal oxygen atom of the R–OO moiety leading to formation of various QOOH radicals are shown in Figure 9. Three distinct 1,5 HATs were identified, where H atoms migrate from the –SH, –OH, and –CH_3_ groups of R1O_2_ to the terminal R–OO moiety. Additionally, a 1,4 HAT path involving a H-shift from the −CH_2_ moiety of R1O_2_ to the terminal R–OO group was identified. These reactions result in the formation of sulfur-, carbon-, and oxygen-centered QOOH radicals. Among these, the 1,5 HAT process via TS25, which produces the S-centered QOOH radical ((CH_3_)_2_C­(OH)­CH­(OOH)­CH_2_S^•^), emerged as the most energetically favorable route. Overall, the findings highlight that HO_2_ elimination via TS20 to form P_20_ ((CH_3_)_2_C­(OH)­CH=CHSH + HO_2_) and intramolecular HAT reactions via TS25, TS26, TS27, and TS28 leading to the formation of P_25_ ((CH_3_)_2_C­(OH)­CH­(OOH)­CH_2_S^•^), P_26_ ((CH_3_)_2_C­(OH)­CH­(OOH)­C^•^HSH), P_27_ ((CH_3_)­C­(^•^CH_2_)­(OH)­CH­(OOH)­CH_2_SH), and P_28_ ((CH_3_)_2_C­(O^•^)­CH­(OOH)­CH_2_SH) are the dominant pathways compared to other possible reaction channels for the R1O_2_ peroxy radical. Previous studies indicate that the QOOH radicals undergo further transformation, leading to the formation of OH radicals and stable cyclic ether products.^52,53^ The QOOH radical can also participate in intramolecular radical attacks, leading to the formation of hydroxyalkyl (HOQO) radicals. However, these pathways generally exhibit higher energy barriers compared to the alternative QOOH decomposition into cyclic ethers and OH radicals.^54^ Therefore, we conducted additional calculations on the S-, C-, and O-centered QOOH radicals formed in the intramolecular HAT reactions associated with the R1O_2_ peroxy radical. Based on the results shown in Figure 9, the S-, C-, and O-centered QOOH radicals (P_25_, P_26_, P_27_, and P_28_) proceed further via TS25a, TS26a, TS27a, and TS28a (with barrier heights of −8.6, −13.9, 4.8, and 27.0 kcal mol^–1^ with respect to the starting R1 + O_2_ reactants) to form the respective cyclic ether products (P_25a_, P_26a_, P_27a_, and P_28a_) and OH radical. The barrier heights for these channels indicate that cyclic ether formation via T26a is predominant compared with other possible paths. The structures of the transition states in Figure 9 clearly indicate O–O bond elongation accompanied by a corresponding C–C–S, C–C–O, and C–C–C angle contraction that enables ring closure to form the cyclic ether and OH radical products. Additionally, the unimolecular decomposition of ^•^QOOH often competes with the bimolecular reaction with O_2_, which produces ^•^OOQOOH. These peroxy radicals can undergo further isomerization and decomposition, resulting in the formation of multiple OH radicals. The reaction pathways P_27_ and P_28_, which lead to the formation of the respective cyclic ether and OH radical products, have high energy barriers and are therefore expected to proceed slowly. Consequently, P_27_ and P_28_ are more likely to react with another O_2_ molecule, which ultimately contributes to the formation of HOMs in the troposphere.

**Figure 9:**
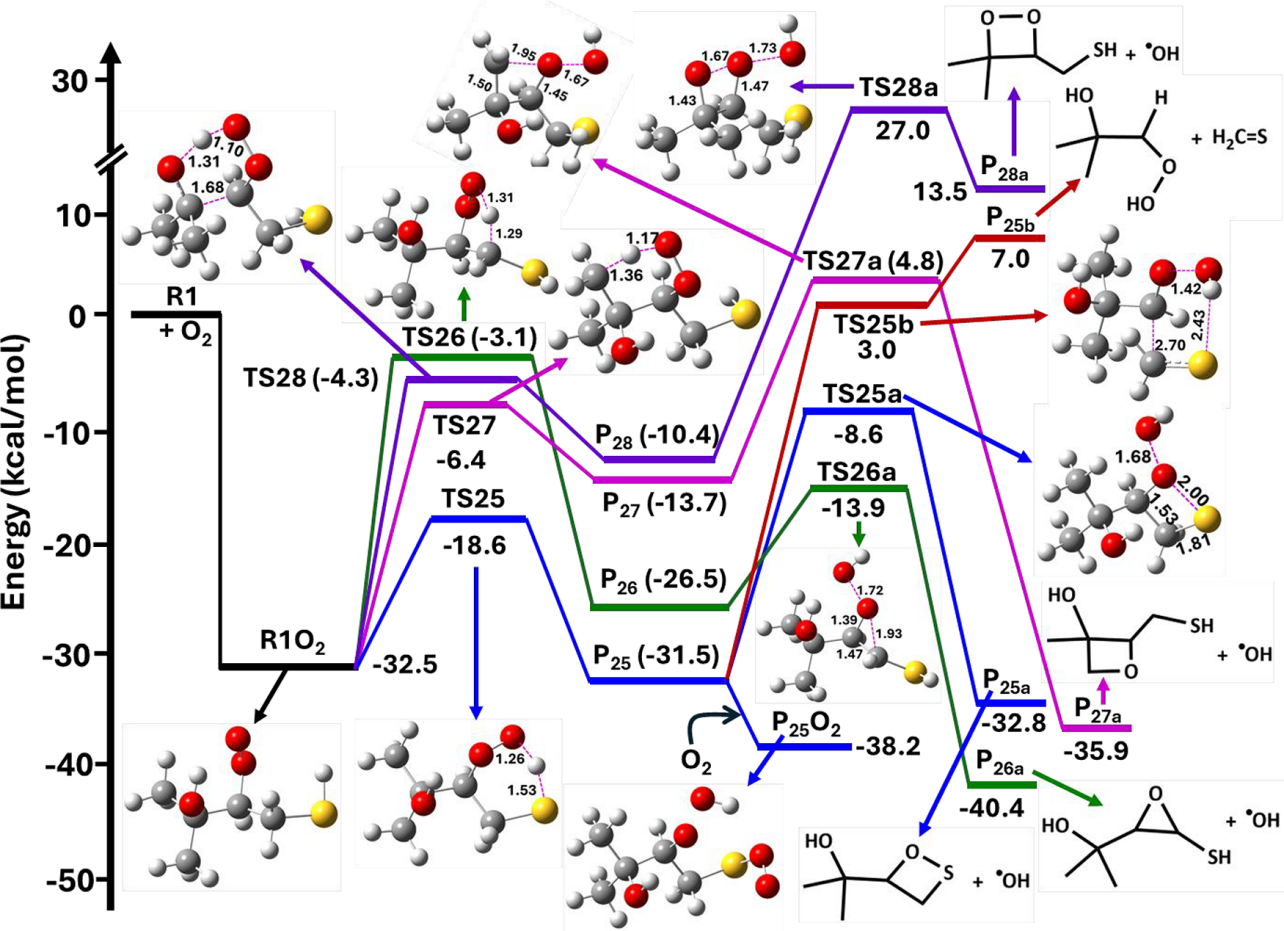
Potential energy profile for the reaction of R1 + O_2_ leading to the R1O_2_ radical, followed by intramolecular HAT to form various QOOH radicals, which then lead to the respective cyclic ethers and OH radial products, calculated at the RHF-RCCSD­(T)-F12a/VDZ-F12//M06-2X/aug-cc-pV­(T+d)­Z level. R1O_2_ = (CH_3_)_2_C­(OH)­CH­(OO^•^)­CH_2_SH; TS25–TS28, TS25a-TS28a, TS25b = transition states; P_25_–P_28_ = QOOH radicals; P_25a_–P_28a_ = cyclic ether + OH radical products; and P_25_O_2_ = (CH_3_)_2_C­(OH)­CH­(OOH)­CH_2_S­(OO^•^).

We performed calculations for the reaction P_25_ → CH_2_S + HOC­(CH_3_)_2_C^•^H­(OOH) at the RHF-RCCSD­(T)-F12a/VDZ-F12//M06-2X/aug-cc-pV­(T+d)­Z level of theory. The PES profile for this reaction is also shown in Figure 9. As depicted, the barrier height for CH_2_S elimination via TS25b, leading to the formation of (CH_3_)_2_C­(OH)­C^•^H­(OOH), is 3.0 kcal mol^–1^ above the energy of the R1 + O_2_ starting reactants. This barrier is ∼11.6 kcal mol^–1^ higher than that for the competing P_25_ → P_25a_ reaction via TS25a. Therefore, this pathway is expected to be significantly slower.

### 3.3 Unimolecular Reactions of the R2O_2_ Radical

The potential energy profile diagram for the reaction of C-centered radical R2 with O_2_ leading to the formation of the R2O_2_ peroxy radical, followed by HO_2_ elimination, obtained at the RHF-RCCSD­(T)-F12a/VDZ-F12//M06-2X/aug-cc-pV­(T+d)­Z level of theory, is shown in Figure 10. The energies of all of the minima and transition states on the PES profiles were calculated relative to the energy of the separated R2 + O_2_ starting reactants. The results in Figure 10 indicate that the C-centered radical R2 primarily interacts with O_2_ to form a barrierless R2O_2_ radical adduct, which possesses 36.7 kcal mol^–1^ of energy. This energy could initiate additional unimolecular reactions. Similar to the case for the R1O_2_ radical, the fate of the R2O_2_ radical adduct also depends on the relative energetics of elimination and intramolecular HAT reactions. Various possible elimination and HAT reactions associated with the R2O_2_ radical adduct are provided in Figure 11. Based on the figure, two distinct unimolecular HO_2_ elimination paths are possible, resulting in the formation of P_29_ (CH_2_)=C­(CH_3_)­CH­(OH)­CH_2_SH + HO_2_) and P_30_ ((CH_3_)_2_C=C­(OH)­CH_2_SH + HO_2_) via eqs 31 and 32, respectively. The schematic representation of the PES profiles for direct HO_2_ elimination reactions involving the R2O_2_ radical is shown in Figure 10. The fully optimized geometries of two different transition states and product complexes are listed in Figures 10 and S3. We identified two types of direct HO_2_ eliminations via TS29 and TS30 from the R2O_2_ radical (see Figure 10). The structures of the transition states indicate that elimination of HO_2_ proceeds through five-membered-ring transition states (TS29 and TS30). These reactions further lead to the respective product complexes (PC6 and PC7) and then to the products P_29_ and P_30_, respectively. The relative barrier heights on the PES profiles indicate HO_2_ elimination via TS29 to be a major path due to its barrier being ∼3.9 kcal mol^–1^ lower compared to other possible HO_2_ eliminations via TS30. It is worth noting that elimination of H_2_O and H_2_S from the R2O_2_ radical is also possible. However, these reaction pathways were not considered because similar reactions in the R1O_2_ radical suggest that these processes have high energy barriers and are not feasible under tropospheric conditions.

**Figure 10:**
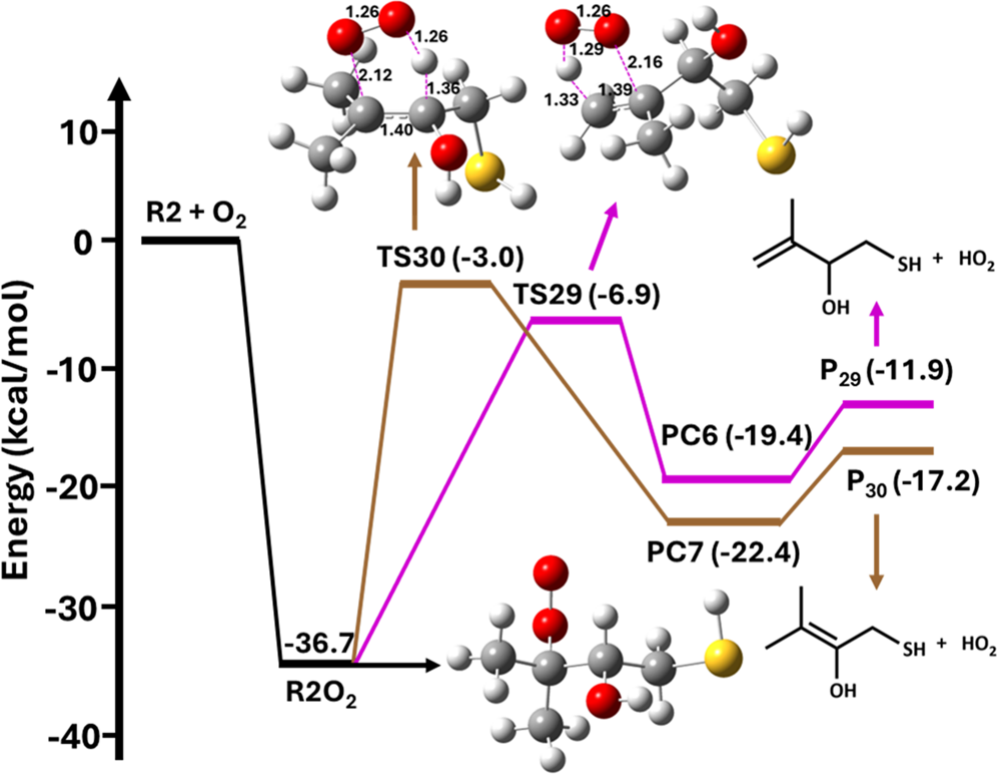
Potential energy profile for the R2 + O_2_ reaction leading to formation of the R2O_2_ radical which then undergoes two different HO_2_ eliminations to form the respective products, calculated at the RHF-RCCSD­(T)-F12a/VDZ-F12//M06-2X/aug-cc-pV­(T+d)­Z level. R2O_2_ = (CH_3_)_2_C­(OO^•^)­CH­(OH)­CH_2_SH; TS29 and TS30 = transition states; PC6 and PC7 = product complexes; and P_29_ and P_30_ = products.

**Figure 11:**
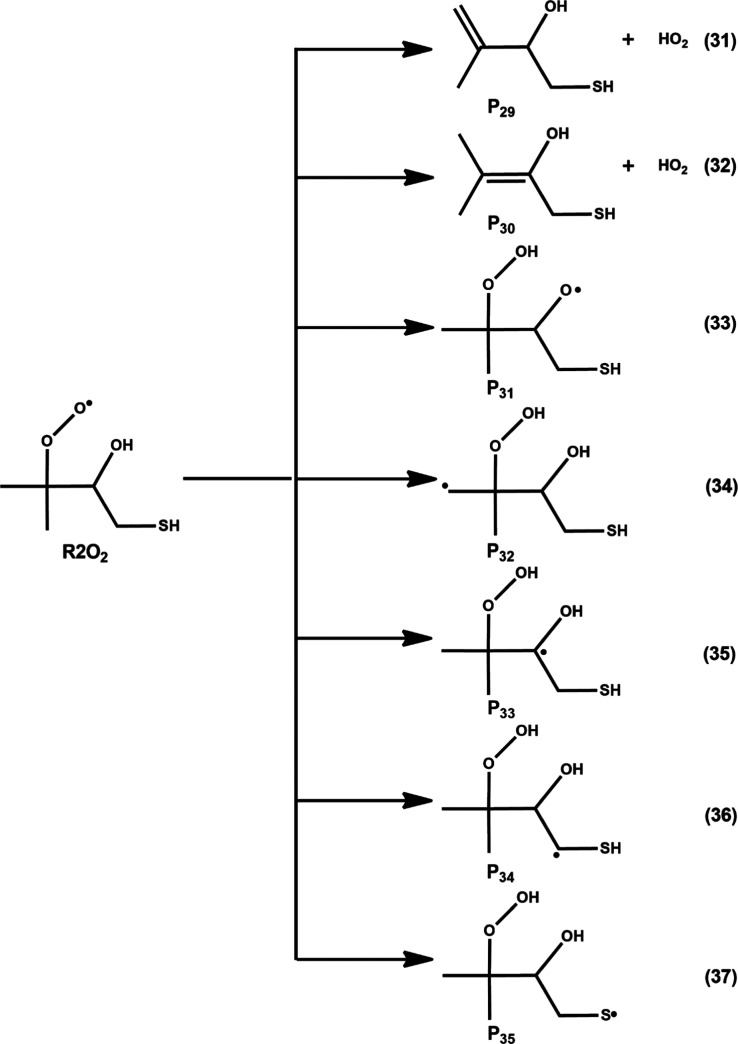
Various possible elimination and HAT pathways for R2O_2_ from the R2 + O_2_ reaction. Equations 31 and 32 represent HO_2_ elimination paths, and eqs 33–37 show HAT reactions. P_29_–P_35_ represent products.

The fate of the R2O_2_ radical also depends on intramolecular HAT reactions. Various possible intramolecular HAT reactions involving R2O_2_ are depicted in Figure 11. According to the figure, a H atom from the –OH, –CH_3_, –CH, –CH_2_, and –SH moieties of R2O_2_ radical is shifted to the terminal oxygen atom of the R–OO group, leading to formation of the corresponding oxygen-, carbon-, and sulfur-centered QOOH radicals via eqs 33–37, respectively. The PES profiles involving all of the stationary points associated with these intramolecular HAT reactions are shown in Figure 12. The optimized structures of the transition states and various possible QOOH products and their subsequent reaction transition states and products are shown in Figures 12 and S3, respectively. According to the figures, all of the H-shift reaction paths can occur through 1,4, 1,5, or 1,6 H-shifts (see TS31–TS35) leading to the formation of various possible carbon-, oxygen-, and sulfur-centered QOOH radical products (see P_31_, P_32_, P_33_, P_34_, and P_35_). The results suggest that the barrier heights for all possible intramolecular HAT reactions are below those of the R2 + O_2_ starting reactants. For example, the intramolecular HAT from the –SH group of R2O_2_ to the terminal oxygen atom of R–OO via TS35, with a barrier height of −18.3 kcal mol^–1^ leads to the formation of an S-centered QOOH radical ((CH_3_)_2_C­(OOH)­CH­(OH)­CH_2_S^•^) product. This reaction is the more dominant one, as its barrier is ∼5.7–17.3 kcal mol^–1^ lower compared to the values of all other possible HAT reactions. Based on the results, direct HO_2_ eliminations and intramolecular HAT-channels are energetically feasible and would be important under atmospheric conditions. Additionally, we further investigated the fate of various possible QOOH radicals associated with the R2O_2_ radical adduct. The obtained stationary points on the PES are also shown in Figure 12. The results indicate that the formed QOOH radicals further proceed to eliminate the OH radical through transition states TS31a, TS32a, TS33a, TS34a, and TS35a with barrier heights of 24.8, −9.8, −19.6, −7.7, and −14.6 kcal mol^–1^, respectively, relative to the energy of the R2 + O_2_ starting reactants. These transition states further lead to the formation of OH radicals along with the respective three-, four-, and five-membered cyclic ether and oxathiolane products such as P_31a_, P_32a_, P_33a_, P_34a_, and P_35a_. The barrier heights for these channels suggest that formation of the three-membered cyclic ether via T33a is the most favorable pathway compared to the other alternatives.

**Figure 12:**
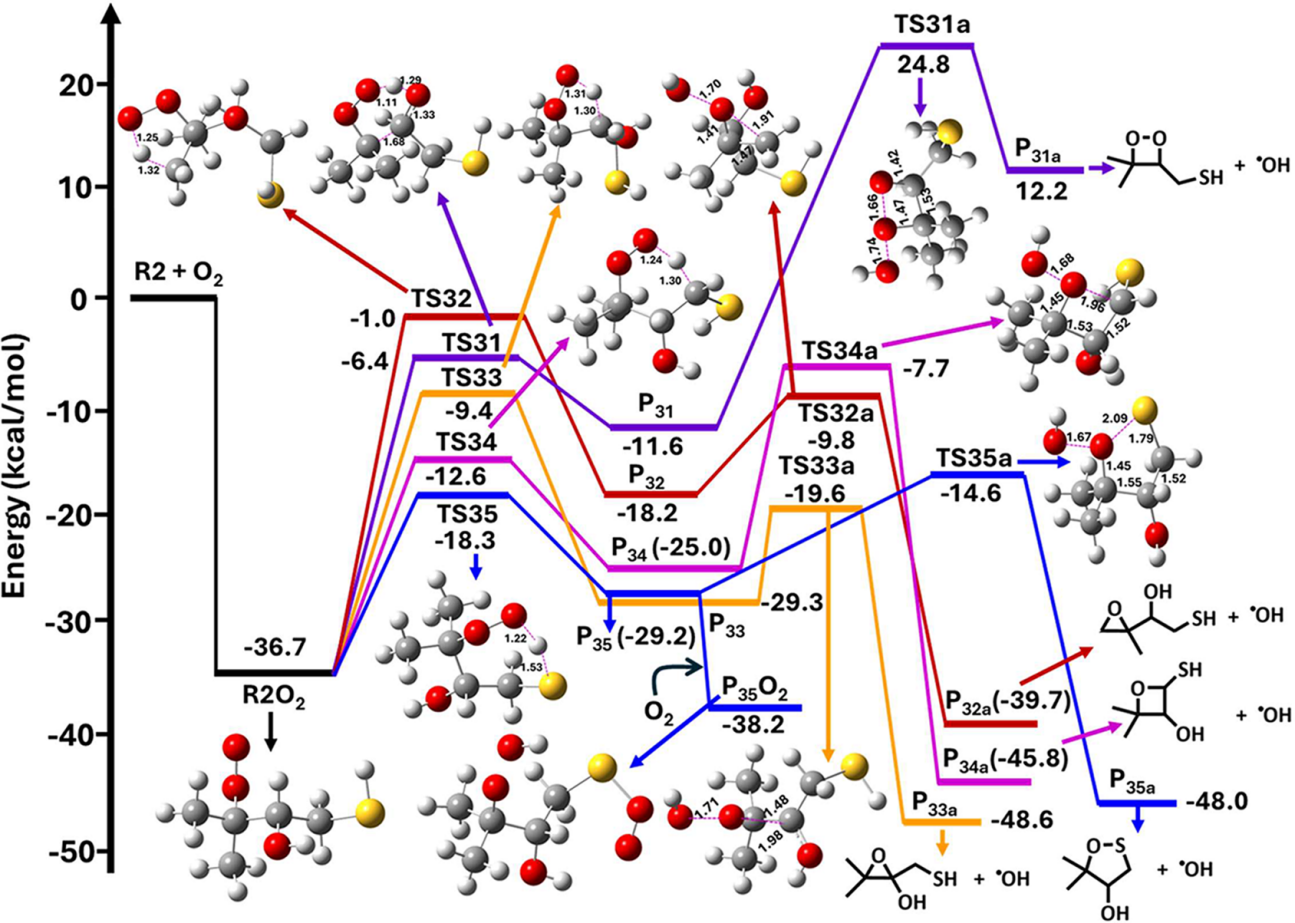
Potential energy profile for the R2 + O_2_ reaction leading to the formation of the R2O_2_ radical, which undergoes intramolecular HATs followed by cyclization to form the respective cyclic ether and oxathiolane products along with the OH radical, calculated at the RHF-RCCSD­(T)-F12A/cc-pVDZ-F12//M06-2X/aug-cc-pV­(T+d)­Z level. The symbols R2O_2_ = (CH_3_)_2_C­(OO^•^)­CHOHCH_2_SH; TS31–TS35 and TS31a–TS35a = transition states; P_31_–P_35_ = QOOH radicals; P_31a_–P_35a_ = cyclic ethers + OH radical products; and P_35_O_2_ = (CH_3_)_2_C­(OOH)­CH­(OH)­CH_2_S­(OO^•^).

The enthalpy and Gibbs free energy at 298 K were determined for all stationary points on the PESs of the R1 + O_2_ and R2 + O_2_ reactions using the RHF-RCCSD­(T)-F12a/VDZ-F12//M06-2X/aug-cc-pV­(T+d)­Z level of theory. These thermodynamic parameters, listed in Tables S1 and S2, are reported relative to the respective R1 + O_2_ and R2 + O_2_ as starting reactants. Based on the enthalpy and Gibbs free energy data in Table S1 for the R1 + O_2_ reaction, the dominant 1,5-HAT pathway through TS25 to form P_25_ ((CH_3_)_2_C­(OH)­CH­(OOH)­CH_2_S^•^) is exothermic and spontaneous, with enthalpy and Gibbs free energy changes of −32.7 and −20.4 kcal mol^–1^, respectively. The formed P_25_ leads to the corresponding cyclic ether and an OH radical. The reaction is exothermic and spontaneous with corresponding enthalpy and Gibbs free energy changes of −33.2 and −30.5 kcal mol^–1^, respectively. Similarly, the enthalpy and Gibbs free energy data presented in Table S2 for the R2 + O_2_ reaction indicates that the dominant 1,6-HAT pathway via TS35 leading to the formation of P_35_ is both exothermic and spontaneous, with enthalpy and Gibbs free energy changes of −30.5 and −17.7 kcal mol^–1^, respectively. The resulting P_35_ further undergoes a reaction to form its corresponding cyclic ether and an OH radical. This subsequent reaction is also exothermic and spontaneous, with enthalpy and Gibbs free energy changes of −48.7 and −44.3 kcal mol^–1^, respectively. The exothermicity and spontaneity of other possible reactions can be understood based on their corresponding enthalpy and Gibbs free energy values provided in Tables S1 and S2 for the R1 + O_2_ and R2 + O_2_ reactions.

### 3.4 Kinetics for the Dissociation of R1 and R2 Radicals

To assess the importance under atmospheric conditions of isomerization and dissociation reactions of carbon-centered R1 and R2 radicals formed from the initial elementary reactions of MBT + OH radical reactants, we calculated rate coefficients for the more dominant channels that were revealed in the present work. Thus, we determined the rate coefficient for the major dissociation channel involving R1 to form ^•^SH + P_5_ via TS5 at 298 K and 1 atm pressure. Similarly, the unimolecular rate coefficient was calculated for the major reaction involving R2 to form P_16_ via TS16 under the same temperature and pressure conditions. With the RRKM method, the obtained rate coefficient values for these reactions via TS5 and TS16 to form ^•^SH + P_5_ and P_16_ were estimated to be 3.0 × 10^5^ and 2.3 × 10^7^ s^–1^, respectively (see MESMER output files). Additionally, rate coefficient calculations were performed for the bimolecular reactions of C-centered R1 and R2 radicals with O_2_ at 298 K. The pseudo-first-order rate coefficients for the reactions R1 + O_2_ → R1O_2_ and R2 + O_2_ → R2O_2_ were found to be 3.0 × 10^7^ for both reactions at 298 K. These values were obtained using an O_2_ concentration of 5.0 × 10^18^ molecules cm^–3^. The results indicate that O_2_ addition to R1 is a factor of ∼100 faster than the dissociation of R1 to P_5_ + ^•^SH. However, we found that the O_2_ addition to R2 and isomerization of R2 to form P_16_ proceed at nearly the same rate. The branching ratio calculations indicate that the addition of O_2_ to the R1 radical to form R1O_2_ accounts for 99% of the reaction at 298 K, while the remaining 1% corresponds to the dissociation of R1 to form ^•^SH and P_5_. In the case of R2, the branching ratio for the addition of O_2_ to R2 to form R2O_2_ is 56.6%, while the isomerization to form P_16_ constitutes 43.4% at the same temperature (298 K). Thus, the C-centered radicals R1 and R2 have a significant opportunity to react with O_2_ in the atmosphere. The formed S-centered radical product P_16_ ((CH_3_)_2_CHCH­(OH)­CH_2_S^•^) further undergoes O_2_ addition at the S atom to form the corresponding RO_2_ radical. This may autooxidize and/or react with NO and HO_2_ radicals under atmospherically relevant conditions. Consequently, these reactions may be a potential source for organosulfates in the atmosphere.

RRKM-ME calculations were performed for the reactions R1 → MBT + ^•^OH (eq 3) and R2 → MBT + ^•^OH (eq 12). These calculations were carried out to evaluate the competition between the formation of MBT + ^•^OH from the R1 and R2 radicals versus their unimolecular decomposition or their reaction with O_2_ under tropospheric conditions. The rate coefficients obtained at 298 K for the reactions R1 → MBT + ^•^OH and R2 → MBT + ^•^OH were 3.3 × 10^–7^ s^–1^ and 6.1 × 10^–6^ s^–1^, respectively. These results indicate that the formation of MBT + ^•^OH from the R1 radical is 11 to 13 orders of magnitude slower than its unimolecular dissociation to form P_5_ + ^•^SH and its reaction with O_2_. Similarly, the formation of MBT + ^•^OH from the R2 radical is 12 orders of magnitude slower than its unimolecular dissociation to form P_16_ and its reaction with O_2_.

It is noted that C-centered R1 and R2 radicals are likely formed with high internal energy following the MBT + ^•^OH reaction. In this work, we assumed thermalized R1 and R2 species for RRKM-ME analysis. A more rigorous treatment would consider chemically activated species (R1*, R2*) and their competing pathways for collisional stabilization versus unimolecular decomposition.

### 3.5 Kinetics for the Reaction of R1 + O_2_

The rate coefficients for the reaction of R1 + O_2_ to form the R1O_2_ radical, followed by various possible elimination and intramolecular HAT reactions, were calculated in the temperatures between 200 and 300 K. The obtained values are provided in Table S3. It is important to note that elimination reactions via TS22, TS23, and TS24 have large barriers, and therefore, these reactions were not considered for rate coefficient calculations in this work. The data in Table S3 indicate that the rate coefficient for the reaction of R1 + O_2_ → R1O_2_ → (CH_3_)_2_C­(OH)­CH=CHSH + HO_2_ via TS20 was 6.9 × 10^–17^ cm^3^ molecule^–1^ s^–1^ at 298 K. Additionally, the rate coefficient for the reaction of R1 + O_2_ → R1O_2_ → (CH_3_)_2_C­(OH)­C­(=O)­CH_2_SH + ^•^OH via TS21 was 6.6 × 10^–22^ cm^3^ molecule^–1^ s^–1^ at the same 298 K. This suggests that the rate coefficient for the HO_2_ elimination via TS20 is ∼5 orders of magnitude higher compared to the OH elimination via TS21. Additionally, the rate coefficients for intramolecular HAT paths were calculated over the same temperature range. The results in Table S3 indicate that the rate coefficient for H atom transfer from the –SH group via TS25 (R1 + O_2_ → R1O_2_ → (CH_3_)_2_C­(OH)­CH­(OOH)­CH_2_S^•^) is 5.8 × 10^–12^ cm^3^ molecule^–1^ s^–1^ at 298 K. The other possible H atom transfers via TS26 (R1 + O_2_ → R1O_2_ → (CH_3_)_2_C­(OH)­CH­(OOH)­C^•^HSH), TS27 (R1 + O_2_ → R1O_2_ → (CH_3_)­C­(^•^CH_2_)­(OH)­CH­(OOH)­CH_2_SH), and TS28 (R1 + O_2_ → R1O_2_ → (CH_3_)_2_C­(O^•^)­CH­(OOH)­CH_2_SH) were estimated to be 9.4 × 10^–17^, 8.8 × 10^–16^, and 2.1 × 10^–16^ cm^3^ molecule^–1^ s^–1^, respectively, at the same 298 K temperature. These results clearly indicate that a H atom transfer from the –SH group of R1O_2_ to the terminal oxygen atom of the R–OO moiety to form an S-centered QOOH radical ((CH_3_)_2_C­(OH)­CH­(OOH)­CH_2_S^•^) via TS25 is more dominant by ∼3–9 orders of magnitude compared to other possible elimination and intramolecular HAT paths at 298 K. This is mainly due to the barrier for this reaction via TS25 being ∼12.2–26.7 kcal mol^–1^ smaller than those of the other possible elimination and HAT paths (see Figures 7 and 9).

### 3.6 Kinetics for the Reaction of R2 + O_2_

The bimolecular rate coefficients for the R2 + O_2_ reaction to form the R2O_2_ radical, followed by subsequent unimolecular elimination and intramolecular HAT reactions, calculated in the temperatures between 200 and 300 K, are displayed in Table S4. The data in the table indicate that the rate coefficient for the major HO_2_ elimination path via TS29 is 1.6 × 10^–16^ cm^3^ molecule^–1^ s^–1^ at 298 K. This value is higher (by 1 order of magnitude) than that of the alternative HO_2_ elimination path via TS30, which has a rate coefficient of 7.1 × 10^–18^ cm^3^ molecule^–1^ s^–1^ at the same temperature. Similarly, the rate coefficients for the intramolecular HAT paths in Table S4 suggest that a H atom shift from the –SH group of R2O_2_ via TS35 to form P_35_ is 5.8 × 10^–13^ cm^3^ molecule^–1^ s^–1^ at 298 K, which is approximately 41 times higher than the next most dominant HAT reaction via TS34 to form P_34_, which has a rate coefficient of 1.4 × 10^–14^ cm^3^ molecule^–1^ s^–1^ at the same temperature. The rate coefficients for other possible intramolecular HAT reactions via TS31, TS32, and TS33 to form P_31_, P_32_, and P_33_ at 298 K were found to be 2.1 × 10^–17^, 2.9 × 10^–19^, and 7.9 × 10^–16^ cm^3^ molecule^–1^ s^–1^, respectively. This suggests that the most dominant HAT reaction rate coefficient via TS35 is ∼4, 6, and 2 orders of magnitude larger when compared to the rate coefficient values obtained for the HAT reactions via TS31, TS32, and TS33 at the same temperature. This is mainly due to the barrier height for the HAT path via TS35 being ∼5.7–17.3 kcal mol^–1^ lower compared to those of the other possible HO_2_ elimination and HAT pathways (see Figures 10 and 12).

### 3.7 Reaction of R1O_2_ and R2O_2_ with the NO/HO_2_ Radical

The unimolecular decomposition of RO_2_ radicals frequently competes with their bimolecular interactions involving the NO and HO_2_ radicals. Consequently, the reactions of R1O_2_ and R2O_2_ with NO and HO_2_ are expected to be potential pathways leading to the formation of the corresponding alkoxy radicals and hydroperoxides, respectively. For example, typical rate coefficients for RO_2_ + NO of 9.0 × 10^–12^ cm^3^ molecule^–1^ s^–1^ and RO_2_ + HO_2_ radical of 1.7 × 10^–11^ cm^3^ molecule^–1^ s^–1^ are well established.^11–13^ Considering the concentrations of NO (∼100 ppt) and HO_2_ radical (∼40 ppt) commonly found in remote pristine environments, indoor settings, or even urban areas during the afternoon,^55–58^ the calculated pseudo-first-order rate coefficients for a typical RO_2_ radical reacting with NO and HO_2_ radical are approximately 2.3 × 10^–2^ s^–1^ and 1.7 × 10^–2^ s^–1^, respectively.^13^ We also determined the unimolecular R1O_2_ reaction rate coefficients (in s^–1^) for all of the reaction paths, and the values obtained at 298 K are given in Table 1. The results indicate that the first-order rate coefficient for the reaction of R1O_2_ → (CH_3_)_2_C­(OH)­C­(=O)­CH_2_SH + ^•^OH, proceeding through TS21, is 3.9 × 10^–13^ s^–1^. This reveals that this pathway occurs slowly by ∼11 orders of magnitude compared to the RO_2_ + NO and RO_2_ + HO_2_ reactions. Furthermore, the first-order rate coefficient for HO_2_ elimination through TS20, to form (CH_3_)_2_C­(OH)­CH=CHSH, was found to be 1.0 × 10^–8^ s^–1^, which is ∼6 orders of magnitude smaller than those of the RO_2_ + NO and RO_2_ + HO_2_ reactions. Additionally, the first-order rate coefficients for intramolecular HATs from the –SH, –CH_2_, –CH_3_, and –OH groups of the R1O_2_ radical via TS25, TS26, TS27, and TS28, leading to sulfur-, carbon-, and oxygen-centered QOOH radicals, were estimated to be 1.0 × 10^2^, 2.2 × 10^–5^, 1.0 × 10^–5^, and 9.8 × 10^–8^ s^–1^, respectively. These findings indicate that the HAT from the –SH group of the R1O_2_ radical via TS25 to form the S-centered QOOH radical is ∼3 orders of magnitude more dominant compared to the bimolecular reactions of RO_2_ + NO and RO_2_ + HO_2_ reactions under tropospheric conditions. In comparison, HAT from the –CH_2_, –CH_3_, and –OH groups of R1O_2_ to form C- and O-centered QOOH radicals was found to be ∼3–6 orders of magnitude slower compared to the bimolecular reactions of R1O_2_ with NO and HO_2_ radical.

**Table 1:** The First-Order Rate Coefficients (in s^–1^) for the Various Elimination and Intramolecular H-Atom Transfer Paths Associated with the R1O_2_ and R2O_2_ Reaction Systems, Calculated at 298 K

TSs	R1O_2_	TSs	R2O_2_
TS20^a^	1.0 × 10^–8^	TS29^a^	3.0 × 10^–9^
TS21^a^	3.9 × 10^–13^	TS30^a^	2.6 × 10^–10^
TS25^b^	1.0 × 10^2^	TS31^a^	1.2 × 10^–9^
TS26^a^	2.2 × 10^–5^	TS32^a^	4.0 × 10^–11^
TS27^a^	1.0 × 10^–5^	TS33^a^	3.5 × 10^–5^
TS28^a^	9.8 × 10^–8^	TS34^a^	1.3 × 10^–3^
		TS35^b^	1.5 × 10^2^

a Rate coefficients were calculated using the RRKM-ME method.

b Rate coefficients were calculated using the MC-TST method.

Similarly, the unimolecular rate coefficients for two different HO_2_ eliminations from the R2O_2_ reaction via TS29 and TS30, leading to formation of P_29_ and P_30,_ were calculated to be 3.0 × 10^–9^ and 2.6 × 10^–10^ s^–1^, respectively. This indicates that HO_2_ elimination reactions via TS29 and TS30 are ∼6 and 8 orders of magnitude (respectively) lower than those for the RO_2_ + NO and RO_2_ + HO_2_ reactions. The unimolecular rate coefficients for H atom transfers from the –OH, –CH_3_, –CH, –CH_2_, and –SH moieties of R2O_2_ via TS31, TS32, TS33, TS34, and TS35 to form the corresponding oxygen-, carbon-, and sulfur-centered QOOH radicals were calculated to be 1.2 × 10^–9^, 4.0 × 10^–11^, 3.5 × 10^–5^, 1.3 × 10^–3^, and 1.5 × 10^2^ s^–1^, respectively. Among these, H atom transfer from the –OH and –CH_3_ groups to form O- and C-centered QOOH radicals via TS31 and TS32 was found to be ∼7–8 orders of magnitude slower compared to those for the RO_2_ + NO and RO_2_ + HO_2_ reactions. On the other hand, H atom transfers from the –CH­(OH) and –CH_2_ to form the corresponding QOOH radical, were found to be slower by 1–2 orders of magnitude, respectively, than for the RO_2_ + NO and RO_2_ + HO_2_ reactions. We found that the HAT from the –SH group of R2O_2_ to form the corresponding S-centered QOOH radical via TS35 was more dominant by ∼3 orders of magnitude when compared to the bimolecular reactions with NO and HO_2_ radicals. Therefore, the intramolecular HAT reactions of R1O_2_ and R2O_2_ mainly proceed via TS25 and TS35 under 100 ppt NO and 40 ppt HO_2_ conditions.

We have performed calculations for the reaction P_25_ + O_2_ → P_25_O_2_, and the corresponding PES profile obtained at the RHF-RCCSD­(T)-F12A/cc-pVDZ-F12//M06-2X/aug-cc-pV­(T+d)­Z level of theory is shown in Figure 9. According to the figure, P_25_ predominantly reacts with O_2_ to form the P_25_O_2_ radical adduct without an energy barrier, and this adduct has an energy of −38.2 kcal mol^–1^. We carried out RRKM-ME calculations to examine the competition between the unimolecular isomerization of P_25_ leading to P_25a_ (cyclic ether + OH radical) formation and the bimolecular reaction of P_25_ with O_2_ forming P_25_O_2_. The calculated rate coefficient for the P_25_ → cyclic ether + OH radical reaction is ∼3.5 × 10^–4^ s^–1^ at 298 K, whereas the pseudo-first-order rate coefficient for the P_25_ + O_2_ → P_25_O_2_ reaction was found to be 1.4 × 10^5^ s^–1^ at the same temperature. These results clearly indicate that P_25_O_2_ formation is significantly faster by 4 × 10^8^ times than the formation of the cyclic ether + OH radical products from P_25_. To determine the pseudo-first-order rate coefficient for this reaction, the O_2_ concentration was set at 5.0 × 10^18^ molecules cm^–3^.

Additionally, we investigated the reaction P_35_ + O_2_ → P_35_O_2_, and the corresponding PES profile is shown in Figure 12. The figure indicates that P_35_ mainly reacts with O_2_ to yield a R_35_O_2_ radical adduct through a barrierless pathway, with the adduct having an energy of −38.2 kcal mol^–1^. We also estimated the rate coefficients to evaluate the competition between the unimolecular isomerization of P_35_ leading to the formation of P_35a_ (cyclic ether + OH radical) via TS35a, and the bimolecular addition of O_2_ to form P_35_O_2_. The unimolecular rate coefficient for the formation of the cyclic ether + OH radical via TS35a was found to be 1.9 × 10^2^ s^–1^ at 298 K, whereas the pseudo-first-order rate coefficient for the P_35_ + O_2_ → P_35_O_2_ reaction was found to be 4.2 × 10^5^ s^–1^ at the same temperature. These results suggest that O_2_ addition is approximately 3 orders of magnitude faster than the unimolecular isomerization of P_35_ leading to the formation of P_35a_. In calculating the pseudo-first-order rate coefficient for this reaction, an O_2_ concentration of 5.0 × 10^18^ molecules cm^–3^ was used.

The reaction of R1O_2_/R2O_2_ radicals with NO and HO_2_ radicals leads to the formation of alkoxy radicals and hydroperoxides, respectively. Building on this, we extended our investigation to explore the behavior of alkoxy radicals produced from the interaction of R1O_2_ and R2O_2_ with NO. Specifically, we examined the subsequent reactions of the alkoxy radicals (CH_3_)_2_C­(OH)­CH­(O^•^)­CH_2_SH (R1O^•^) and (CH_3_)_2_C­(O^•^)­CH­(OH)­CH_2_SH (R2O^•^) generated from the R1O_2_ + NO and R2O_2_ + NO reactions. The unimolecular rate coefficients for R1O_2_ and R2O_2_ via TS25 and TS35 were calculated to be 1.0 × 10^2^ and 1.5 × 10^2^ s^–1^ at 298 K, respectively. These values indicate that unimolecular pathways are significantly faster by 3 orders of magnitude than the corresponding bimolecular reactions that would lead to the formation of R1O^•^ and R2O^•^ at concentrations of NO of ∼100 ppt. As such, the formation of R1O^•^ and R2O^•^ through bimolecular channels is unlikely to be competitive under atmospheric conditions. Nonetheless, we have included these minor reaction pathways for the sake of completeness, as R1O^•^ and R2O^•^ species may still form when NO concentrations reach tens of ppb in the morning within polluted urban atmospheres or within indoor environments after cooking.^13,59,60^ Under such high NO conditions, the bimolecular reactions of R1O_2_ and R2O_2_ with NO predominantly yield alkoxy radicals (R1O^•^ and R2O^•^). Therefore, it is worthwhile to consider the subsequent transformations of R1O^•^ and R2O^•^.

The PES profiles involving various stationary points for the R1O radical dissociation were calculated at the RHF-RCCSD­(T)-F12a/VDZ-F12//M06-2X/aug-cc-pV­(T+d)­Z level and are shown in Figure S4. The results indicate that the R1O radical undergoes two different C–C bond cleavages via TS36 and TS37, leading to the formation of (CH_3_)_2_C^•^(OH) + HC­(=O)­CH_2_SH and (CH_3_)_2_C­(OH)­C­(=O)H + ^•^CH_2_SH products, respectively. The barrier heights for the formation of TS36 and TS37 were estimated to be 3.1 and 7.4 kcal mol^–1^ above that of the R1O radical. Additionally, intramolecular H atom transfer from the –SH group to the oxygen atom of the R1-O radical, leading to the formation of the (CH_3_)_2_C­(OH)­CH­(OH)­CH_2_S^•^ product, was also studied. This occurs because the H atom in the –SH group is more labile than the H atoms in the –OH, –CH, –CH_3_, and –CH_2_ groups. The barrier height for the formation of the transition state TS38 was found to be 10.1 kcal mol^–1^. This indicates that the formation of (CH_3_)_2_C^•^(OH) + HC­(=O)­CH_2_SH products from the R1O radical is the dominant reaction compared with the other possibilities.

The PES profiles for the various possible dissociation reactions for the R2O radical, calculated at the RHF-RCCSD­(T)-F12a/VDZ-F12//M06-2X/aug-cc-pV­(T+d)­Z level, are shown in Figure S5. According to the figure, the R2O radical also undergoes two different C–C bond cleavages via TS39 and TS40, with barrier heights of 5.1 and 16.3 kcal mol^–1^ above the R2O radical. The reaction paths then lead to the formation of acetone + HC^•^(OH)­CH_2_SH and ^•^CH_3_ + CH_3_C­(=O)­CH­(OH)­CH_2_SH products, respectively. The reaction path via intramolecular H atom transfer from the –SH group to the oxygen atom of the R2-O radical, leading to the formation of the (CH_3_)_2_C­(OH)­CH­(OH)­CH_2_S^•^ product, was also studied. The barrier height for this reaction via TS41 was found to be −1.9 kcal mol^–1^ below that of the R2O radical starting reactant. This low barrier is mainly due to the adoption of a stabilizing six-membered-ring transition state structure. This barrier height is ∼7–18.0 kcal mol^–1^ lower than those for other possible channels. Therefore, the present data suggest that the formation of the (CH_3_)_2_C­(OH)­CH­(OH)­CH_2_S^•^ product from the R2O radical is more dominant compared to others.

Based on the results reported in our previous^9^ and the present work, the most plausible mechanism for the MBT + OH radical reaction is illustrated in Figure 13. It shows that the reaction of the MBT + OH radical leads to the formation of carbon-centered radicals R1 and R2 via addition pathways, indicated by black arrows. The subsequent reactions of the carbon-centered R1 and R2 radicals are shown with blue and pink arrows, respectively. Once released, the R1 radical rapidly reacts with the O_2_ radical under tropospheric conditions to form the corresponding R1O_2_ radical. In the case of R2, it can either undergo O_2_ addition to form R2O_2_ or isomerize to produce the sulfur-centered MBT–OH radical. The energies and kinetics results in the current work indicate that the formed R1O_2_ and R2O_2_ radicals undergo 1,5 and 1,6 H atom shifts from –SH to the terminal oxygen atom of the R–OO group, leading to the formation of S-centered QOOH radicals (see Figure 13). The formed S-centered QOOH radical rapidly interacts with O_2_ leading to the formation of the O_2_QOOH radical. This is because the rate of unimolecular isomerization of S-centered QOOH radicals leading to the formation of a cyclic ether + OH radical was found to be significantly slow under tropospheric conditions. Under high HO_2_ radical atmospheres, the R1O_2_ and R2O_2_ radicals react with HO_2_ radicals to form their respective hydroperoxides (see Figure 13). In the case of highly polluted atmospheric conditions (morning within polluted urban atmospheres or indoor environments after cooking), where the concentrations of NO can reach up to tens of ppb, R1O_2_ and R2O_2_ react with NO to form the corresponding alkoxy radicals (R1O^•^ and R2O^•^). These radicals then undergo C–C bond scission and H atom transfer reactions to produce air pollutants such as HC­(=O)­CH_2_SH, (CH_3_)_2_C­(OH)­C­(=O)­H, CH_3_C­(=O)­CH_3_, and various S- and C-centered alkyl radicals (see Figure 13). In turn, these S- and C-centered radicals rapidly react with atmospheric O_2_, which is followed by subsequent reactions to form various C- and S-containing compounds in the atmosphere.

**Figure 13:**
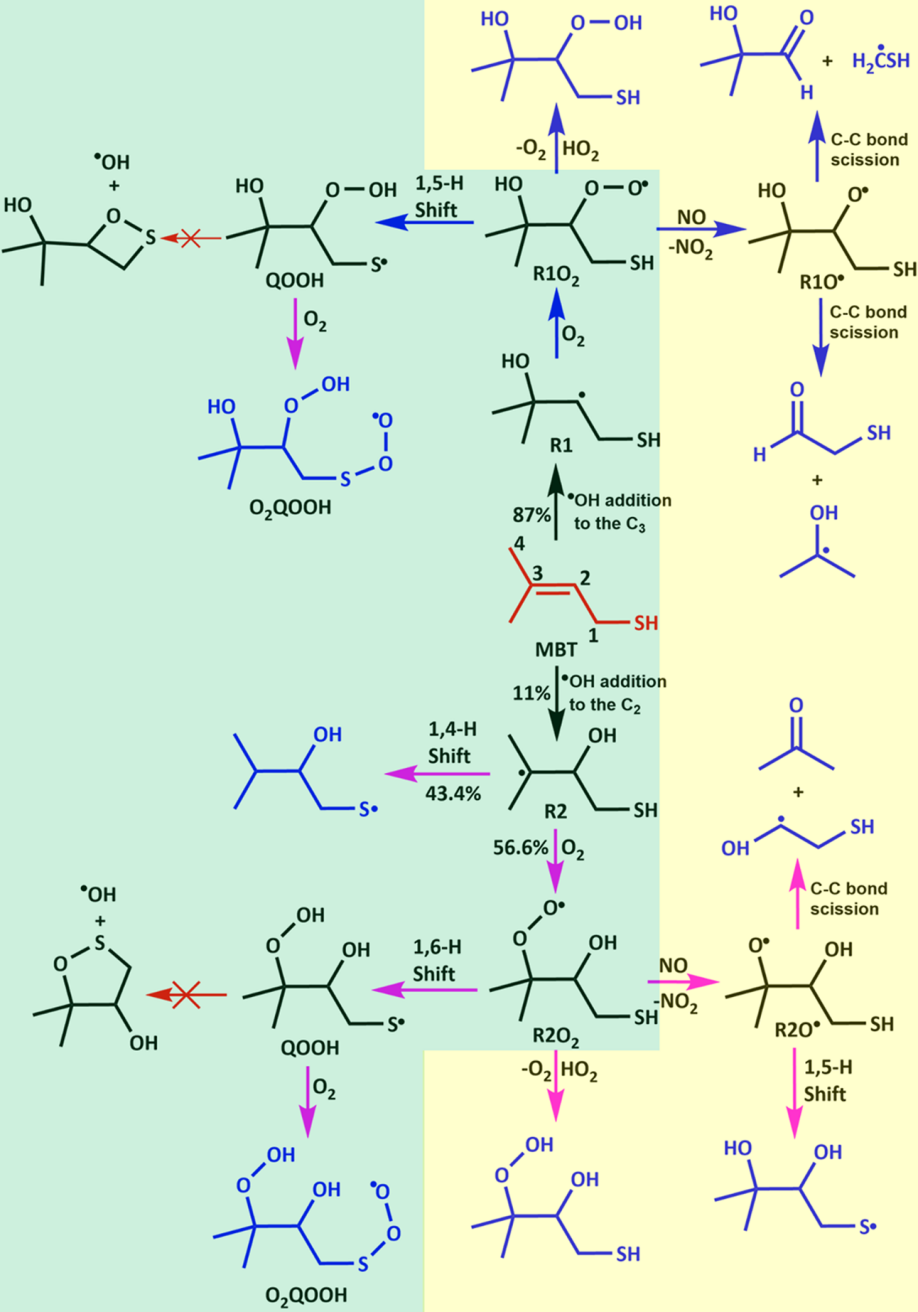
Most plausible mechanism for the transformation of MBT in the presence of OH radical, followed by subsequent reactions of the R1 and R2 radicals with O_2_, the HO_2_ radical, and NO leading to the formation of highly oxygenated peroxy radical products, HC­(O)­CH_2_SH, (CH_3_)_2_C­(OH)­C­(=O)­H, CH_3_C­(O)­CH_3_, and various sulfur- and carbon-centered alkyl radicals. The structures of the starting reactant and final products are shown in red and blue colors, respectively. The reactions of the carbon-centered R1 and R2 radicals with O_2_, followed by their subsequent reactions, are indicated by blue and pink arrows, respectively. Products are shown in blue; major products are set against a green background, and minor products are set against a yellow background.

## 4 Conclusions

The key characteristics of carbon-centered radicals R1 and R2 (generated from the interaction of terrestrial plant-emitted MBT with atmospheric hydroxy radicals), along with their subsequent reactions with atmospheric O_2_, were investigated using both computational calculations and kinetic modeling. This study demonstrates that the self-dissociation of carbon-centered radical R1 proceeds much more slowly than its reaction with O_2_ under atmospheric conditions. Similarly, the carbon-centered radical R2 can either undergo addition with O_2_ to form R2O_2_ or isomerize to produce the sulfur-centered MBT–OH radical. In both cases, the radicals are effectively intercepted by O_2_, leading to the formation of the corresponding peroxy radicals, R1O_2_ and R2O_2_. While H_2_O, the OH radical, and H_2_S elimination pathways are possible for these radicals, these channels were found to be slower when compared to HO_2_ elimination and intramolecular HAT reactions. The most favorable pathway involves H atom transfer from the –SH group of R1O_2_ and R2O_2_ which have transition states located at 18.6 and 18.3 kcal mol^–1^, respectively, below the energy of the separated R1 + O_2_ and R2 + O_2_ reactants. The rate calculations indicate that the intramolecular H atom transfer mechanism from the –SH group of R1O_2_ and R2O_2_ proceeds approximately 3 orders of magnitude faster than their bimolecular reactions with the NO or HO_2_ radical, respectively. Furthermore, the intramolecular HAT from the –SH group to the terminal oxygen atom of R1O_2_ and R2O_2_, leading to formation of two different S-centered QOOH radicals (followed by subsequent reactions), was found to be rapid. The formed S-centered QOOH radicals further react with O_2_ leading to the formation of highly oxygenated peroxy radicals (O_2_QOOH) via an autoxidation mechanism. This work highlights how gas-phase autoxidation of MBT-derived peroxy radicals can act as a direct source of highly oxygenated peroxy radical products, even under moderately polluted atmospheric conditions. These compounds lead to the formation and growth of secondary organic aerosols (SOAs), which influence air quality, climate, and the formation of secondary pollutants.^61^ In highly polluted environments, they also lead to the formation of additional air pollutants, such as HC­(=O)­CH_2_SH, (CH_3_)_2_C­(OH)­C­(=O)­H, CH_3_C­(=O)­CH_3_, and various sulfur- and carbon-centered alkyl radicals. The released sulfur-containing compounds, such as HC­(=O)­CH_2_SH and S-centered radicals, undergo oxidation in the atmosphere ultimately leading to sulfur-containing aerosol formation. These aerosols absorb infrared radiation and reflect solar rays, thereby influencing atmospheric processes. The reflection of incoming light by aerosols contributes to a reduction in global warming.^62,63^

## Supplementary Material



## Data Availability

The output files for all the stationary points involved in the quantum chemical calculations and Mesmer rate calculations are available online (10.5281/zenodo.14782761).

## Notes

Transition state structures of various possible dissociation and isomerization reactions of R1 and R2, and product complexes and product structures for the R1 + O_2_ reaction; optimized structures of product complexes and products involved in R2O_2_ reactions; PES profiles for R1O^•^ and R2O^•^ unimolecular dissociation; enthalpies and free energies of all the stationary points on the PES profiles of R1 + O_2_ and R2 + O_2_ reaction systems; and bimolecular rate coefficients for the reaction of R1 + O_2_ and R2 + O_2_ systems (PDF)
