# Supplementary material for: A Computational Study on the Atmospheric Fate of Carbon-Centered Radicals from the 3‑Methyl-2-butene-1-thiol + •OH Reaction: Mechanistic Insights and Atmospheric Implications
Source: J Phys Chem A. 2025 Jul 18;129(30):6866–82. doi: 10.1021/acs.jpca.5c00743 (PMC12319919; doi:10.1021/acs.jpca.5c00743)
Supplement: Supplementary file 1 [file jp5c00743_si_001.pdf]

Computational Study on the Atmospheric Fate of Carbon-Centered Radicals From the 3-Methyl-2-Butene-1-Thiol + •OH Reaction: Mechanistic Insights and Atmospheric Implications

Parandaman Arathala[a], Avinash Kumar[b] and Rabi A. Musah[a,*]

[a]*Department of Chemistry, Louisiana State University, Baton Rouge, Louisiana 70803, USA*

[b]*Aerosol Physics Laboratory, Physics Unit, Faculty of Engineering and Natural Sciences, Tampere University, 33720 Tampere, Finland*

*Address correspondence to: rmusah@lsu.edu

Supporting Information:

Figures S1 and S2: The transition state structures of various possible dissociation and isomerization reactions of R1 and R2, and product complexes and product structures for the R1 + O_2 reaction; Figure S3: Optimized structures of product complexes and products involved in R2O_2 reactions. Figures S4 and S5: PES profiles for R1O• and R2O• unimolecular dissociation; Tables S1 and S2: Enthalpies and free energies of all the stationary points on the PES profiles of R1 + O_2 and R2 + O_2 reaction systems. Tables S3 and S4: Bimolecular rate coefficients for the R1 + O_2 and R2 + O_2 reaction systems. The output files for all the stationary points involved in the quantum chemical calculations and Mesmer rate calculations are available online (10.5281/zenodo.14782761).

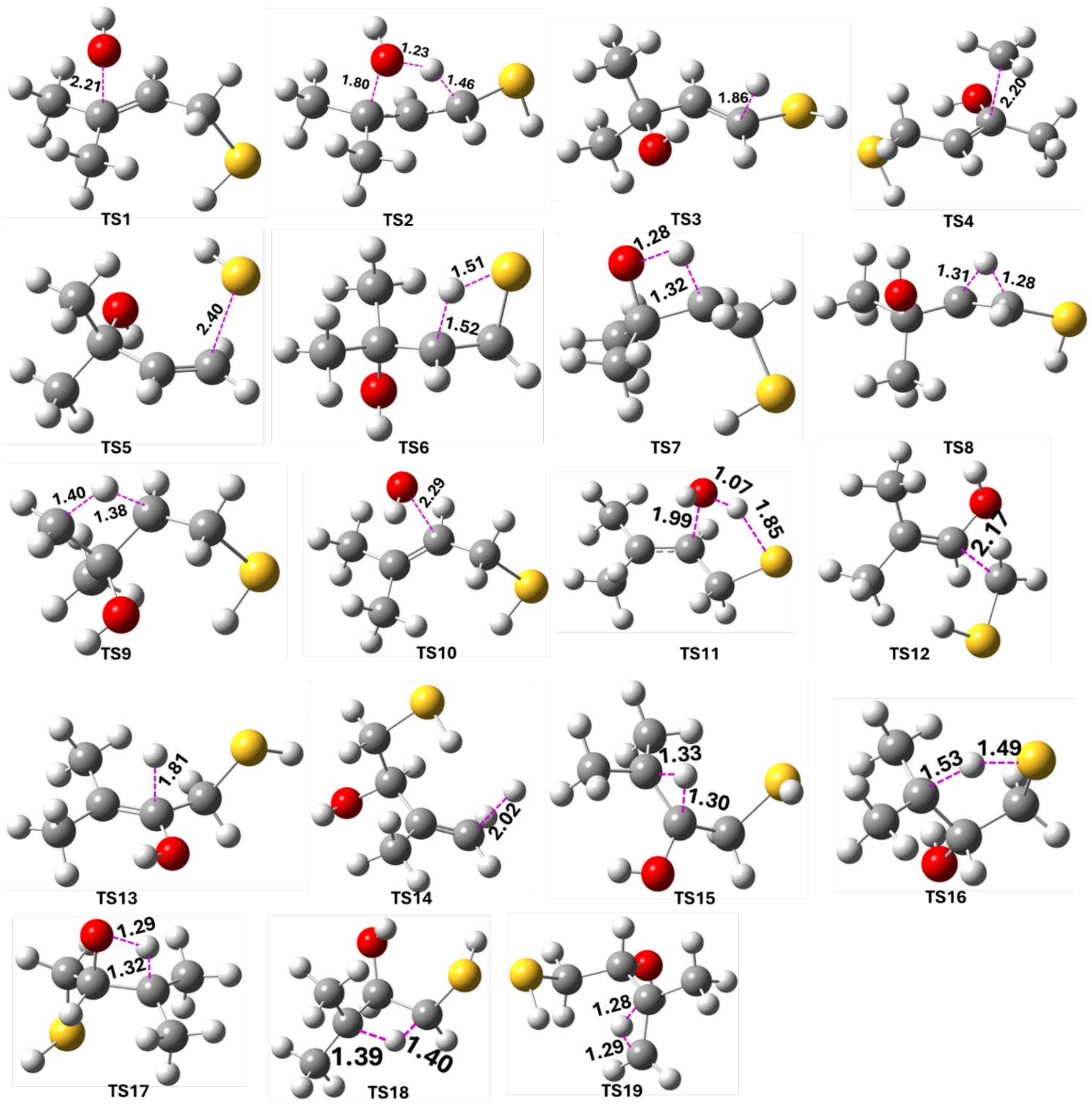

Figure S1. Transition state structures for various possible self-dissociation and isomerization reactions associated with R1 and R2, optimized at the M06-2X/aug-cc-pV(T+d)Z level. Bond lengths are provided in Å units. The symbols for carbon, hydrogen, sulfur, and oxygen are represented with black, white, yellow, and red colors, respectively.

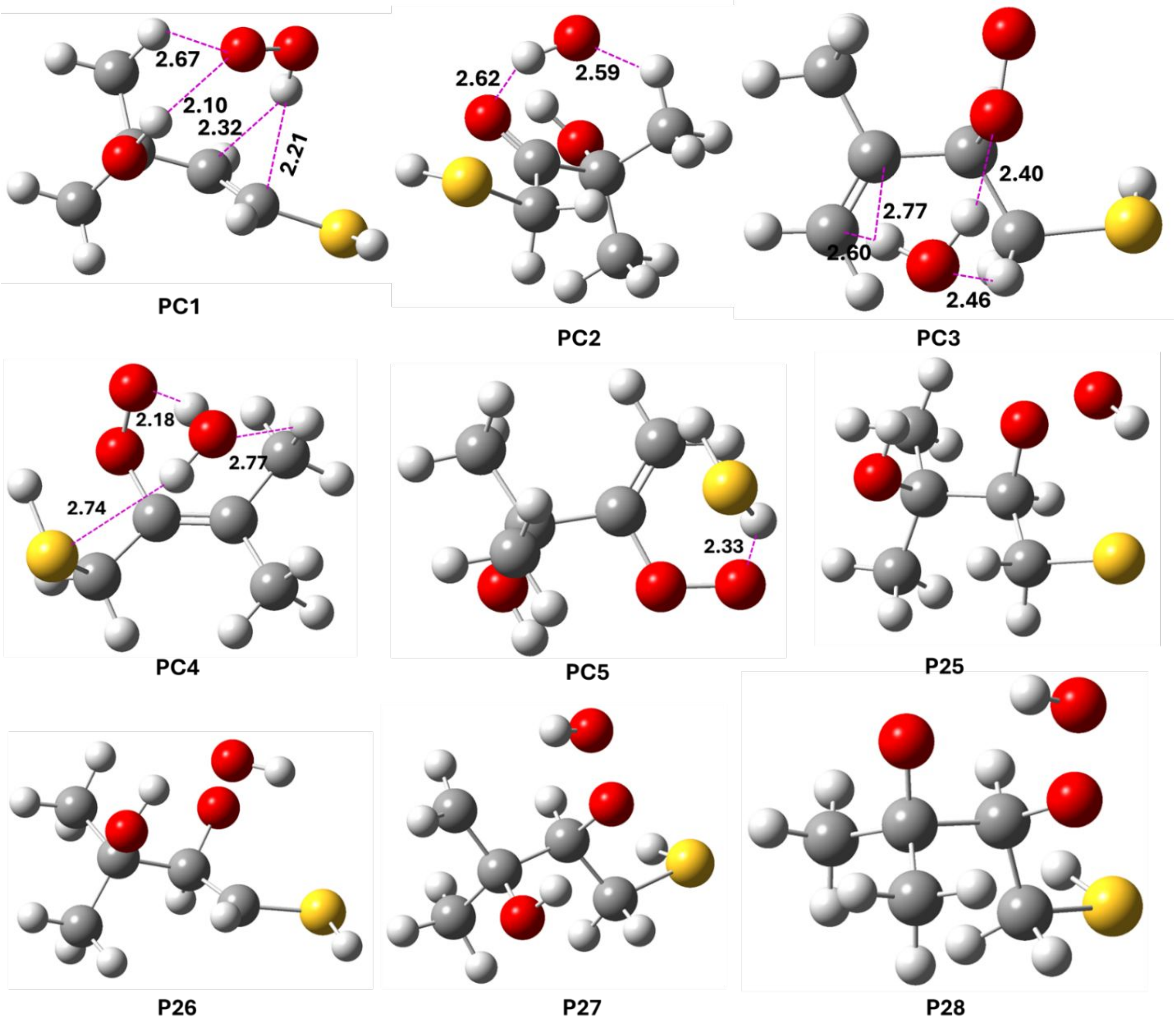

Figure S2. The fully optimized geometries of product complexes and products formed from the reaction of R1 + O_2 obtained at the M06-2X/aug-cc-pV(T+d)Z level of theory. Bond lengths are provided in Å. The symbols for carbon, hydrogen, sulfur, and oxygen are represented by black, white, yellow, and red colors, respectively.

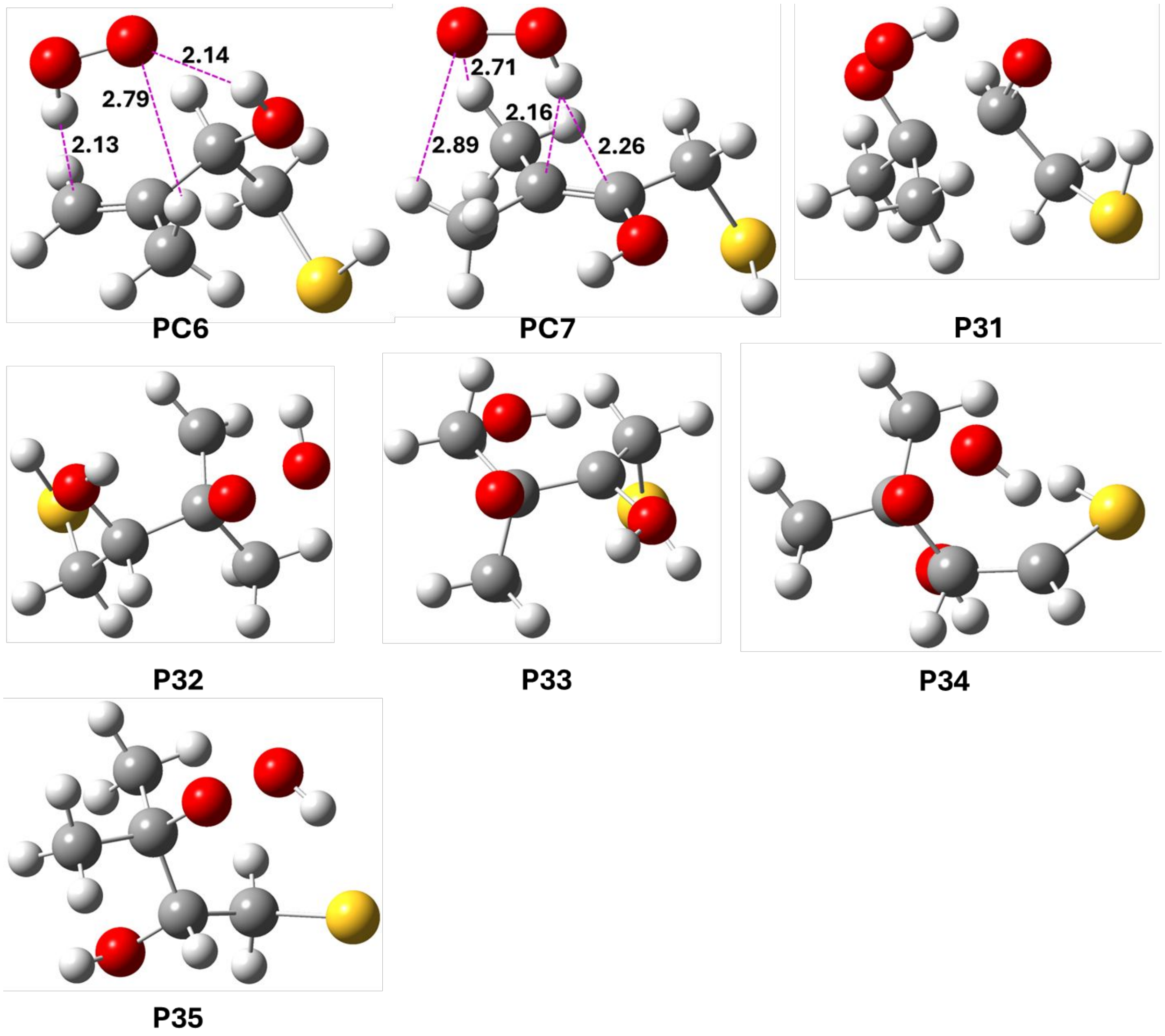

Figure S3. The fully optimized geometries of product complexes and products formed from the reaction of R2 + O_2 obtained at the M06-2X/aug-cc-pV(T+d)Z level of theory. Bond lengths are provided in Å. The symbols for carbon, hydrogen, sulfur, and oxygen are represented by black, white, yellow, and red colors, respectively.

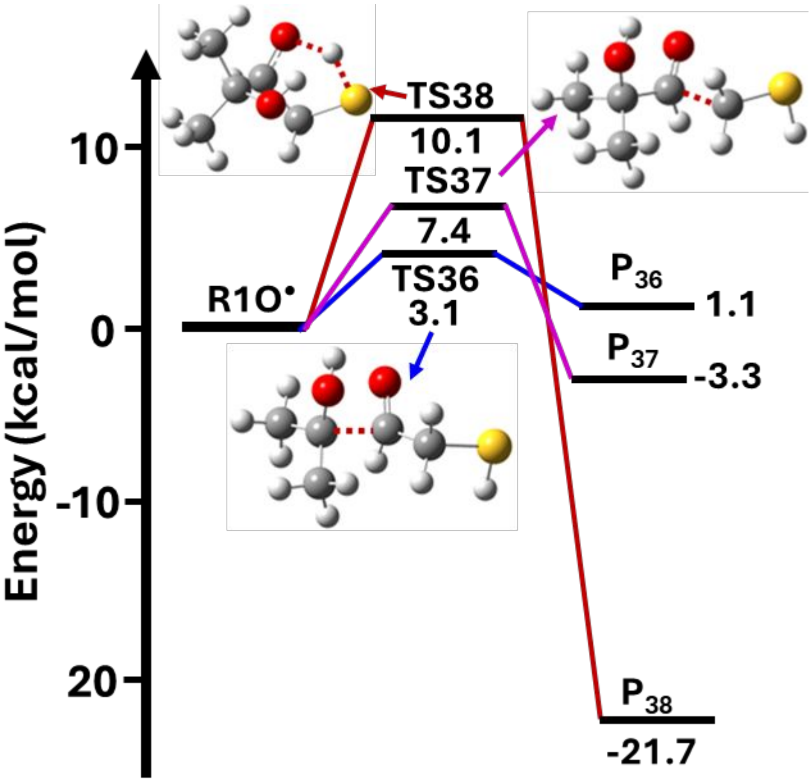

Figure S4: Potential energy profile for the unimolecular dissociation reactions of R1O radical, leading to the formation of the corresponding products, calculated at the RHF-RCCSD(T)-F12A/cc-pVDZ-F12//M06-2X/aug-cc-pV(T+d)Z level of theory. R1O• = $(CH_3)_2C(OH)CHO^{\bullet}CH_2SH$; TS36 – TS38 = transition states; P_{36} = $HC(O)CH_2SH$ + $CH_3C^{\bullet}(OH)CH_3$; P_{37} = $(CH_3)_2C(OH)C(=O)H$ + $^{\bullet}CH_2SH$; and P_{38} = $(CH_3)_2C(OH)CH(OH)CH_2S^{\bullet}$.

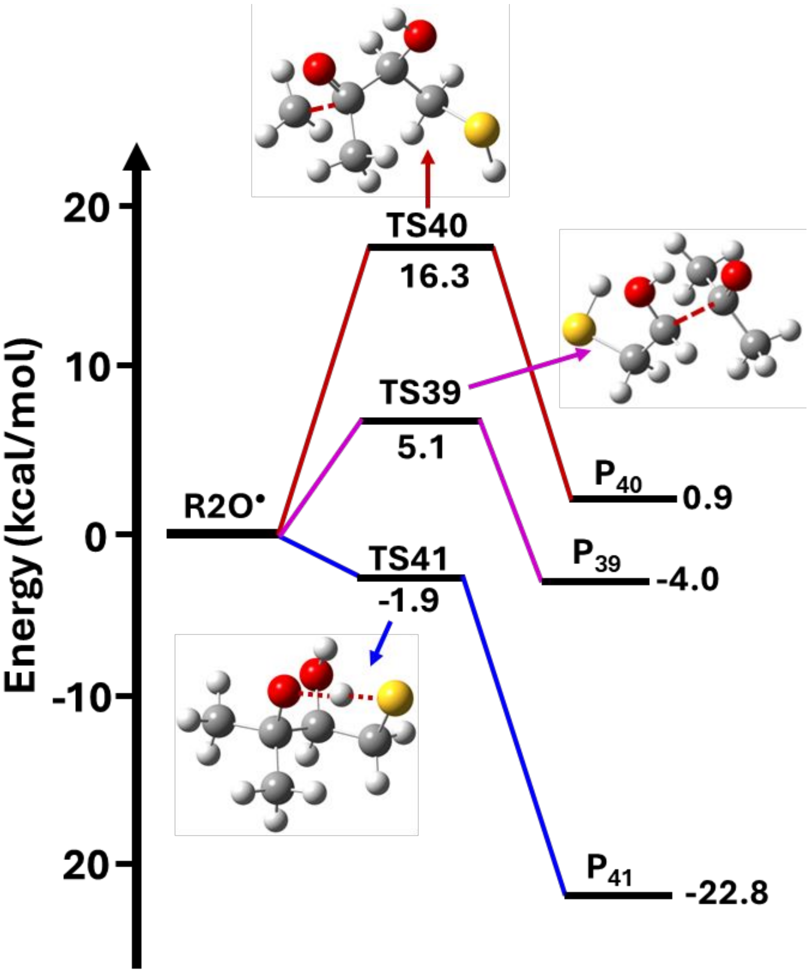

Figure S5: Potential energy profile for the unimolecular dissociation reactions of R2O radical, leading to the formation of the corresponding products, calculated at the RHF-RCCSD(T)-F12A/cc-pVDZ-F12//M06-2X/aug-cc-pV(T+d)Z level of theory. R2O$^{\bullet}$ = $(CH_3)_2C(O^{\bullet})CH(OH)CH_2SH$; TS39 – TS41 = transition states; P_{39} = $CH_3C(O)CH_3$ + $HC^{\bullet}(OH)CH_2SH$; P_{40} = $CH_3C(=O)CH(OH)CH_2SH$ + $^{\bullet}CH_3$; and P_{41} = $(CH_3)_2C(OH)CH(OH)CH_2S^{\bullet}$.

**Table S1.** Enthalpies (ΔH (298 K)) and Gibbs free energies (ΔG (298K)) of various stationary points involved in the reaction of R1 with O_2, calculated at the RHF-RCCSD(T)-F12a/VDZ-F12//M06-2X/aug-cc-pV(T+d)Z level.[a]		
**Stationary point**	**Enthalpy (kcal mol^{-1})**	**Free energy (kcal mol^{-1})**
R1 + O_2	0.0	0.0
R1O_2	-33.7	-21.3
TS20	-4.3	7.8
PC1	-21.7	-12.6
P_{20}	-14.7	-15.6
TS21	6.9	19.3
P_{21}	-63.5	62.2
TS22	33.9	45.8
PC3	-20.1	-12.2
P_{22}	-17.2	-17.7
TS23	37.4	48.8
PC4	-23.4	-14.7
P_{23}	-21.4	-22.3
TS24	40.9	52.0
PC5	-15.9	-7.7
P_{24}	-14.6	-14.6
TS25	-20.2	-6.9
P_{25}	-32.7	-20.4
TS25a	-9.8	2.6
P_{25a}	-33.2	-30.5
TS25b	2.6	12.6
P_{25b}	7.1	5.4
TS26	-4.6	8.4
P_{26}	-27.2	-15.7
TS26a	-14.7	-3.1
P_{26a}	-40.4	-38.3
TS27	-8.1	5.2
P_{27}	-14.6	-2.9
TS27a	3.9	15.7
P_{27a}	-36.1	-33.9
TS28	-5.6	6.7
P_{28}	-11.4	0.5
TS28a	26.1	37.7
P_{28a}	13.6	15.1

[a]The enthalpic (*H*) and free energy (*G*) corrections were derived from M06-2X/aug-cc-pV(T+d)Z level calculations.

**Table S2.** Enthalpies (ΔH (298 K)) and Gibbs free energies (ΔG (298K)) of various stationary points involved in the reaction of R2 with O_2, calculated at the RHF-RCCSD(T)-F12a/VDZ-F12//M06-2X/aug-cc-pV(T+d)Z level.[a]		
**stationary point**	**enthalpy (kcal mol^{-1})**	**Free energy (kcal mol^{-1})**
R2 + O_2	0.0	0.0
R2O_2	-38.0	-24.9
TS29	-8.4	5.0
PC6	-19.9	-9.6
P_{29}	-12.2	-12.0
TS30	-4.1	8.5
PC7	-22.4	-13.7
P_{30}	-17.3	-17.7
TS31	-8.4	6.2
P_{31}	-13.0	0.5
TS31a	23.4	36.8
P_{31a}	11.8	15.3
TS32	-2.9	11.8
P_{32}	-19.3	-6.2
TS32a	-10.9	1.9
P_{32a}	-40.3	-36.6
TS33	-11.2	3.3
P_{33}	-30.4	-17.7
TS33a	-20.4	-8.8
P_{33a}	-48.6	-46.5
TS34	-14.3	0.0
P_{34}	-26.1	-13.1
TS34a	-8.8	4.3
P_{34a}	-46.2	-42.7
TS35	-20.2	-5.5
P_{35}	-30.5	-17.7
TS35a	-16.0	-2.6
P_{35a}	-48.7	-44.3
[a]The enthalpic (*H*) and free energy (*G*) corrections were derived from M06-2X/aug-cc-pV(T+d)Z level calculations.		

**Table S3.** Bimolecular rate coefficients (cm^3 $molecule^{-1}$ s^{-1}) for the various possible elimination and intramolecular HAT reactions of the $R1O_2$ radical associated with the R1 + O_2 reaction in the temperatures between 200 and 300 K.						
**T(K)**	**TS20**	**TS21**	**TS25**	**TS26**	**TS27**	**TS28**
**200**	2.3×10^{-18}	5.4×10^{-24}	4.7×10^{-12}	8.5×10^{-18}	8.8×10^{-17}	1.0×10^{-17}
**220**	4.7×10^{-18}	1.3×10^{-23}	5.1×10^{-12}	1.4×10^{-17}	1.4×10^{-16}	1.9×10^{-17}
**240**	9.7×10^{-17}	3.4×10^{-23}	5.4×10^{-12}	2.2×10^{-17}	2.3×10^{-16}	3.6×10^{-17}
**260**	1.9×10^{-17}	9.3×10^{-23}	5.6×10^{-12}	3.6×10^{-17}	3.6×10^{-16}	6.6×10^{-17}
**280**	3.8×10^{-17}	2.6×10^{-22}	5.7×10^{-12}	6.0×10^{-17}	5.8×10^{-16}	1.2×10^{-16}
**298**	6.9×10^{-17}	6.6×10^{-22}	5.8×10^{-12}	9.4×10^{-17}	8.8×10^{-16}	2.1×10^{-16}
**300**	7.3×10^{-17}	7.3×10^{-22}	5.8×10^{-12}	9.8×10^{-17}	9.2×10^{-16}	2.2×10^{-16}

**Table S4.** Bimolecular rate coefficients (cm^3 $molecule^{-1}$ s^{-1}) for the various possible elimination and intramolecular HAT reactions of the $R2O_2$ radical associated with the R2 + O_2 reaction in the temperatures between 200 and 300 K.							
**T(K)**	**TS29**	**TS30**	**TS31**	**TS32**	**TS33**	**TS34**	**TS35**
**200**	1.1×10^{-17}	2.5×10^{-16}	1.8×10^{-18}	1.8×10^{-20}	1.2×10^{-16}	2.8×10^{-15}	1.8×10^{-13}
**220**	1.9×10^{-17}	1.6×10^{-16}	2.9×10^{-18}	3.1×10^{-20}	1.7×10^{-16}	3.8×10^{-15}	2.3×10^{-13}
**240**	3.3×10^{-17}	5.7×10^{-17}	4.9×10^{-18}	5.5×10^{-20}	2.5×10^{-16}	5.4×10^{-15}	2.9×10^{-13}
**260**	5.7×10^{-17}	5.8×10^{-18}	8.1×10^{-18}	9.6×10^{-20}	3.7×10^{-16}	7.5×10^{-15}	3.7×10^{-13}
**280**	9.8×10^{-17}	1.4×10^{-18}	1.3×10^{-17}	1.7×10^{-19}	5.5×10^{-16}	1.0×10^{-14}	4.7×10^{-13}
**298**	1.6×10^{-16}	7.1×10^{-18}	2.1×10^{-17}	2.9×10^{-19}	7.9×10^{-16}	1.4×10^{-14}	5.8×10^{-13}
**300**	1.6×10^{-16}	9.3×10^{-18}	2.2×10^{-17}	3.0×10^{-19}	8.2×10^{-16}	1.5×10^{-14}	5.9×10^{-13}
